# Development of a Validated Rate-Based Model for CO_2_ Absorption in Aqueous 2‑Amino-2-methyl-1-propanol and Piperazine Blends Using Aspen Plus

**DOI:** 10.1021/acs.energyfuels.5c03281

**Published:** 2025-09-29

**Authors:** Diego Morlando, Ying Zhang, Shu Wang, Hanna K. Knuutila

**Affiliations:** a Department of Chemical Engineering, 8018 Norwegian University of Science and Technology, Trondheim N-7491, Norway; b AspenTech Shanghai, Pudong, Shanghai 201210, China; c 113186 Aspen Technology, Inc., Bedford, Massachusetts 01730, United States

## Abstract

In this work, we developed a new e-NRTL thermodynamic framework for CO_2_ absorption in aqueous mixtures of 2-amino-2-methyl-1-propanol (AMP) and piperazine (PZ) in Aspen Plus. The e-NRTL AMP/PZ/H_2_O/CO_2_ model was fitted on experimental data covering a range of AMP concentration from 12 to 48 mass % and PZ concentration from 2 to 26 mass %, temperature from 20 to 160 °C and CO_2_ loading from 0 to 1.03 $\frac{mol_{CO_{2}}}{mol_{AMP}+mol_{PZ}}$ . The model predicts the CO_2_ solubility, as partial pressure of CO_2_, over aqueous AMP/PZ solutions within an absolute average relative deviation (AARD) of 26.3%, the total pressure of the system with an AARD value of 7.0%, the heat of absorption of CO_2_ with an AARD value of 10.2%, and the estimated free CO_2_ concentration with an AARD value of 13.1%. The model gives a good representation of liquid speciation as a function of the CO_2_ loading and amine concentration. The model shows good predictions of the CO_2_ solubility over aqueous AMP/PZ solutions at relevant absorber, stripper and water wash amine concentrations and temperatures. The developed e-NRTL model, in combination with mass transfer and CO_2_ absorption kinetics modeling, is validated with two pilot campaigns: one at the University of Kaiserslautern and one at the Technology Centre of Mongstad. The developed rate-based model predicts the CO_2_ capture, rich loading, and specific reboiler duty within 5% absolute average relative deviation.

## Graphical abstract

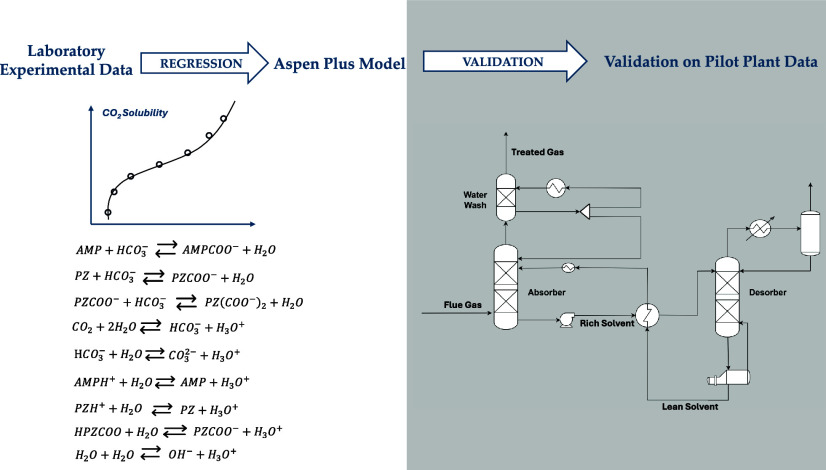

## Introduction

An aqueous mixture of 2-amino-methyl-1-propanol (AMP) and piperazine (PZ) has recently gained attention in the field of post-combustion amine-based CO_2_ capture. The 3 M AMP + 1.5 M PZ aqueous blend, also known as the CESAR1 solvent, was proposed by Feron et al.^1^ as a new benchmark for the amine-based CO_2_ capture process. The CESAR1 solvent has been tested in different pilot plants and shows better energy performance, higher chemical stability, and greater flexibility than the former benchmark, monoethanolamine (MEA).^2^ Recently, Morlando et al.^2^ reviewed the research work on the properties and the use of aqueous AMP/PZ blends for CO_2_ capture and outlined existing experimental and modeling gaps. Some of the experimental gaps were addressed in recent works.^3,4^ In this work, we aim to develop an accurate thermodynamic model for the absorption of CO_2_ in aqueous AMP/PZ solutions in Aspen Plus. The thermodynamic framework of this work uses the e-NRTL (electrolyte Non-Random Two-Liquid) model implemented in Aspen Plus v14 to describe the liquid phase and the Redlich–Kwong equation of state for the gas phase. The e-NRTL framework has already been proven to be accurate in predicting the thermodynamics of different aqueous amine systems with CO_2_.^5,6^ To develop a thermodynamically consistent model for CESAR1, it is essential to accurately represent the thermodynamics of the AMP/H_2_O/CO_2_ and PZ/H_2_O/CO_2_ systems, as CESAR1 is a blend of these two amines. The thermodynamic frameworks for the AMP/H_2_O/CO_2_ and PZ/H_2_O/CO_2_ systems are available in Aspen Plus. The existing PZ/H_2_O/CO_2_ model is considered accurate for the scope of this work, as it will be shown later, but the AMP/H_2_O/CO_2_ is improved based on new recent experimental findings.^2,4^ The regression is carried out using the Data Regression System (DRS) available in Aspen Plus. A sequential regression method is used as suggested by Hilliard.^7^ The developed thermodynamic model is then used, in combination with mass transfer and kinetics models, to develop a process model validated using pilot plant data from the Technology Centre of Mongstad (TCM)^8^ and the University of Kaiserslautern.^9^ Li et al.^10^ and Yi et al.^11^ also developed an e-NRTL-based thermodynamic framework for the AMP-PZ-H_2_O-CO_2_ system in Aspen Plus. The model developed in this work is deemed to provide a better and more comprehensive description of the thermodynamics of the AMP-PZ-H_2_O-CO_2_ system than the previous works, since it includes the new thermodynamic data collected by Morlando et al.^4^ Liquid speciation, CO_2_ physical solubility data, and high-temperature CO_2_ solubility data (up to 150 ^◦^C) were not available for the CESAR1 solvent before the work by Morlando et al.^4^ and could not have been used in the parameter fitting. Furthermore, in this work, the thermodynamic framework is integrated with a reaction and mass transfer model and validated using pilot plant data, which was not done by Li et al.^10^ and Yi et al.^11^

## Thermodynamic Modeling: Theory and Methodology

### Chemistry of the AMP/PZ/H_2_O/CO_2_ System

The reactions describing the AMP/PZ/H_2_O/CO_2_ system are given in eqs 1–10.^2,12^

$$2H_{2}O⇄H_{3}O^{+}+{\mathrm{OH}}^{-}$$ 1

$$2H_{2}O+{\mathrm{CO}}_{2}⇄H_{3}O^{+}+{\mathrm{HCO}}_{3}^{-}$$ 2

$$H_{2}O+{\mathrm{HCO}}_{3}^{-}⇄H_{3}O^{+}+{\mathrm{CO}}_{3}^{2-}$$ 3

$$\mathrm{AMP}+{\mathrm{HCO}}_{3}^{-}⇄{\mathrm{AMPCOO}}^{-}+H_{2}O$$ 4

$$H_{2}O+{\mathrm{AMPH}}^{+}⇄\mathrm{AMP}+H_{3}O^{+}$$ 5

$$H_{2}O+{\mathrm{PZH}}^{+}⇄\mathrm{PZ}+H_{3}O^{+}$$ 6

$$H_{2}O+{\mathrm{PZH}}_{2}^{2+}⇄{\mathrm{PZH}}^{+}+H_{3}O^{+}$$ 7

$$\mathrm{PZ}+{\mathrm{HCO}}_{3}^{-}⇄{\mathrm{PZCOO}}^{-}+H_{2}O$$ 8

$${\mathrm{PZCOO}}^{-}+{\mathrm{HCO}}_{3}^{-}⇄\mathrm{PZ}({\mathrm{COO}}^{-}{)}_{2}+H_{2}O$$ 9

$$H_{2}O+{\mathrm{PZH}}^{+}{\mathrm{CO}}_{2}^{-}⇄{\mathrm{PZCOO}}^{-}+H_{3}O^{+}$$ 10

This reaction network consists of 10 different reactions and 15 species. The model developed in this work is simplified by neglecting the protonation reaction, forming PZH_2_^2+^, eq 7. This is reasonable given its high dissociation constant^13,14^ and consequently, very low concentration in the liquid phase.

The equilibrium of the reactions is defined in terms of Gibbs free energy, eq 11, to ensure consistency among the thermodynamic definition and calculation in Aspen Plus.^7^

$$-\frac{\Delta G_{j}^{0}}{RT}=\ln\left(K_{j}\right)$$ 11

Where Δ*G*_j_^0^ is the change of reference state Gibbs free energy for the reaction *j*, *R* is the universal gas constant, *T* is the system temperature and *K*_j_ is the equilibrium constant of the reaction *j*.

The standard Gibbs formation energy of the aqueous ionic compounds at 298.15 K, Δ*G*_298.15K_^aq,∞^, and the standard formation enthalpy of the aqueous ionic compounds at 298.15 K, Δ*H*_298.15K_^aq,∞^, are used as adjustable parameters in fitting the equilibrium constant and the thermodynamic experimental data.

### Activity Coefficient

The e-NRTL model is an activity-based model, and the activity coefficients, γ, are crucial for thermodynamic properties calculations. All the thermodynamic properties are highly affected by the activity of the species. The activity coefficients are calculated based on the excess Gibbs energy.^15^ The reference states used in this work are pure components for H_2_O, AMP, and PZ molecules. For CO_2_, the infinite dilution reference state in the mixed solvent is used for the vapor–liquid calculation (i.e., Henry’s constant), while an infinite dilution in aqueous solution is used for the chemical equilibria calculations. The reference state for the ionic species is infinite dilutions in aqueous solution. The protonated piperazine carbamate, HPZCOO, is defined as a zwitterion in the current framework. Therefore, HPZCOO can interact with other molecular species through the NRTL parameters. The activity coefficient is calculated as a solute, and therefore, the contribution to the solution enthalpy and Gibbs free energy is calculated as a solute.

The parameters describing the local interaction contribution accounting for the interaction between the molecule-anion-cation are used as adjustable parameters for the activity coefficient of the species. These parameters are a function of three adjustable parameters in the e-NRTL framework, named GMENCC, GMENCD, and GMENCN, respectively. The first two are energy parameters describing the local interaction between molecules (*i*) and electrolytes (*j*). The definition of the local interaction energy parameter, τ_*ij*_, is reported in eq 12. GMENCD defines the functionality of the energy parameters with the temperature. In this work, only the GMENCC are used as adjustable parameters to fit the experimental data, while the GMENCD are set to their default value, 0. GMENCN is also known as a nonrandomness factor, and it is set to 0.2.^15^ Additionally, in this work, the electrolyte-electrolyte binary parameters, GMENCC, are set to their default, 0. Finally, the binary parameters, GMENCC, including H_3_O^+^ and OH^–^, are also set to 0, given their very low concentration compared to the other ionic species.

$$\tau_{ij}=\mathrm{GMENCC}+\frac{\mathrm{GMENCD}}{T}$$ 12

### Thermodynamic Modeling Methodology

As mentioned above, a new AMP/H_2_O/CO_2_ model was developed in this work, while the PZ/H_2_O/CO_2_ model previously developed by AspenTech was validated and found to be adequate for the scope of this work. After that, the AMP/PZ/H_2_O and AMP/PZ/H_2_O/CO_2_ models were developed. For the AMP/H_2_O system, the vapor–liquid equilibrium data from Belabbaci et al.,^16^ Hartono et al.,^17^ Nouacer et al.,^18^ and Pappa et al.,^19^ the heat capacity data from Chen et al.,^20^ Chiu and Li,^21^ Zhang et al.,^22^ and the excess enthalpy data from Mathonat et al.^23^ were used to fit the NRTL parameters between AMP and H_2_O. The fitted NRTL parameters with their standard deviation for the AMP/H_2_O are available in the Supporting Information, Table S1. For the AMP/H_2_O/CO_2_, the CO_2_ solubility data, as CO_2_ partial pressure, *P*_CO_2__, by Hartono et al.,^24^ Li and Chang,^25^ Dash et al.,^26^ and Kundu et al.^27^ and as total pressure, *P*_tot_, by Morlando et al.,^4^ Hartono et al.,^12,24^ and Tong et al.^28^ and the heat of absorption of CO_2_ data by Morlando et al.^4^ have been used in the regression of the energy parameters, of Gibbs formation energy, enthalpy of formation and heat capacity of the AMP ionic species, AMPH^+^/AMPCOO^–^. The speciation data by Ciftja et al.^29^ were not implemented directly in the fitting. The NMR data by Ciftja et al.^29^ for the AMP-carbamate, AMPCOO^–^, were used to fine-tune the equilibrium constant of AMPCOO^–^. The fitted parameters, with their standard deviation, for the AMP/H_2_O/CO_2_ are available in the Supporting Information, Table S2.

For the ternary system, AMP/PZ/H_2_O, the AMP/PZ NRTL parameters were fitted on the volatility data by Hartono et al.^17^ and the recent data published by Charalambous et al.,^30^ and the liquid heat capacity data from Chen et al.^20^ However, the fitted values and standard deviation indicated overparameterization. Therefore, the NRTL parameters for the ternary system were set to 0. This result was also observed by Hartono et al.^12^

For the AMP/PZ/H_2_O/CO_2_, the CO_2_ solubility data from Morlando et al.,^4^ Hartono et al.,^12^ Li et al.,^31^ Dash et al.,^32,33^ and Brúder et al.^34^ were included in the energy parameters regression. The heat of absorption of CO_2_ data, liquid speciation, and CO_2_ physical solubility data from Morlando et al.^4^ have also been included in fitting. The CO_2_ physical solubility data have been expressed in terms of the mole fraction of free CO_2_, obtained via the N_2_O analogy.^35^ The parameters for the Henry’s constant of CO_2_ in pure piperazine were fitted to the CO_2_ physical solubility data for the AMP/PZ/H_2_O/CO_2_ system. In the PZ/H_2_O/CO_2_ model, the CO_2_ Henry’s constant in pure piperazine was assumed to be the same as in water, since data on N_2_O solubility in pure piperazine are missing in the open literature. The fitted Henry’s constant of CO_2_ for pure piperazine did not significantly alter the predictive performance of the PZ/H_2_O/CO_2_ model. The validation results of the PZ/H_2_O/CO_2_ model in this article are obtained from the model with the new Henry’s constant of CO_2_ in pure piperazine parameters. The fitted parameters with their standard deviation for the AMP/PZ/H_2_O/CO_2_ are available in the Supporting Information, Table S3.

In this work, the data sets used in the fitting and the model performance on these data sets are reported. The fitted parameters are available in the Supporting Information, Tables S3 and S4. The availability of the new speciation data, vapor-liquid equilibrium (VLE) data at high temperatures for various AMP and PZ concentrations and the availability of low amine concentration data relevant for the water wash section allowed us to develop a model providing accurate predictions in a temperature range from 20 to 160 °C, in a concentration range of AMP from 0.53 to 48 mass % and of PZ from 0.26 to 26 mass %. The model developed in this work should provide a comprehensive thermodynamic representation of the AMP/PZ/H_2_O/CO_2_ system over a broad range of operative conditions and give more reliable predictions than previous works.

## Process Modeling: Theory and Methodology

### Mass Transfer and CO_2_ Absorption Kinetics

Aspen Plus software allows for the running of rate-based simulations using RateSep, a rate-based unit for multistage separation processes. RateSep requires modeling of the transfer properties such as liquid viscosity, surface tension, thermal conductivity, and binary diffusion. The liquid viscosity has been described according to the Aspen-based liquid viscosity quadratic mixing and the Jones-Dole electrolyte correction model. We fitted the AMP/PZ parameters for the viscosity of the unloaded solution using the data for the CESAR1 blend collected by Morlando et al.^3^. The viscosity of the unloaded CESAR1 solvent is well predicted, as shown in Figure S3. The Jones-Dole electrolyte model does not have any adjustable parameters for amine blends; therefore, the viscosity data for CO_2_-loaded CESAR1 data were not fitted. The liquid molar volume is calculated according to the Clarke model. The density data for CO_2_-loaded and unloaded CESAR1 have been used in the parameters’ estimation. The model parameters are given in Table S6. The model predicts well the density for CESAR1 as shown in Figure S3. Finally, the Nernst–Hartley model together with the Wilke–Chang model is used to calculate the effective binary diffusion coefficients. The diffusivity is an important parameter for mass transfer modeling. The effective diffusivity of an ion is calculated according to eq 13.

$$D_{i}=\left(\frac{RT}{z_{i}F^{2}}\right)\left(l_{1,i}+l_{2,i}T\right)\underset{k}{\sum }x_{k}$$ 13

where *D*_i_ is the effective diffusivity of an ion *i* in a liquid mixture with electrolytes, F is the Faraday’s constant, *z*_*i*_ is the charge number of species *i*, *T* is the system temperature, and *x*_*k*_ mole fraction of any molecular species *k*, *l*_1, *i*_ and *l*_2, *i*_, known as IONMOB, are adjustable parameters. In this work, the default value of 5.0, as proposed by AspenTech, has been used for the PZ and AMP ions.

CO_2_ absorption kinetics plays a crucial role in the simulation of the CO_2_ capture plant, especially for the absorber unit. The protonation and deprotonation reactions were considered in equilibrium and not kinetics-controlled. The reaction rates for the kinetics-controlled reactions are defined in Table 1. The reactions were all defined as activity-based. The AMP carbamation reaction, ID3, has been obtained by Jamal et al.^36^ The reaction rate for the formation of PZ carbamate and dicarbamate, ID5 and ID7, has been obtained by Samanta and Bandyopadhyay.^37^ Finally, the bicarbonate formation reaction parameters have been obtained by Pinsent et al.^38^ The reaction parameters for the reverse reactions (ID2, ID4, ID6, and ID8 in Table 1) have been tuned to satisfy the chemical equilibrium.

**Table 1:** Kinetics Reaction Rates Used in This Work

ID	reactions	concentration basis	pre-exponential factor	**E** $\left[\frac{J}{\mathrm{kmol}}\right]$	Reference
1	CO_2_ + OH^–^ → HCO_3_^–^	activity	1.33 × 10^17^	5.55 × 10^7^	Pinsent et al.^38^
2	HCO_3_^–^ → CO_2_ + OH^–^	activity	6.63 × 10^16^	1.07 × 10^8^	this work
3	AMP + CO_2_ + H_2_O → AMPCOO^–^ + H_3_O^+^	activity	1.38 × 10^10^	2.04 × 10^7^	Jamal et al.^36^
4	AMPCOO^–^ + H_3_O^+^ → AMP + CO_2_ + H_2_O	activity	6.14 × 10^24^	6.17 × 10^7^	this work
5	PZ + CO_2_ + H_2_O → PZCOO^–^ + H_3_O^+^	activity	3.99 × 10^10^	2.53 × 10^6^	Samanta and Bandyopadhyay^37^
6	PZCOO^–^ + H_3_O^+^ → PZ + CO_2_ + H_2_O	activity	1.26 × 10^24^	6.17 × 10^7^	this work
7	PZCOO^–^ + CO_2_ + H_2_O → PZ(COO^–^)_2_ + H_3_O^+^	activity	3.21 × 10^14^	3.52 × 10^7^	Samanta and Bandyopadhyay^37^
8	PZ(COO^–^)_2_ + H_3_O^+^ → PZCOO^–^ + CO_2_ + H_2_O	activity	8.53 × 10^17^	1.05 × 10^7^	this work

### Process Modeling Methodology

The process model validation has been carried out by simulating the absorber and stripper as single units. This validation approach allows a more robust analysis of the process unit performance, minimizing error propagation between the two units. This approach has been used to validate several rate-based models for CO_2_ capture using amine-based solvents.^39–42^

The experimental flue gas temperature, pressure, composition, mass flow rate, and the lean stream temperature, pressure, composition, and mass flow rate are the required inputs for the absorber simulation. The geometry of the absorber and the packing material used are also required inputs for the simulation. The simulated rich stream and outlet gas temperature, mass flow rate, and composition are used to validate the model. Furthermore, the simulated temperature profile and CO_2_ liquid concentration in the absorber are compared with the experimental data when available.

The experimental rich stream temperature and pressure, composition and mass flow rate, pressure of the stripper, condenser temperature and pressure, and reboiler duty are the required inputs for the stripper simulation. The geometry of the stripper and the packing material used are also required inputs for the simulation. The simulated lean stream and outlet gas stream mass flow rate and composition are used to validate the model. Furthermore, the simulated temperature profiles in the stripper are compared to the experimental data when available.

Table 2 reports the submodules and parameters used for the process model validation. They are the same for both the pilot campaigns simulated in this work. The liquid and vapor mass transfer were modeled according to Bravo et al. (1985).^43^ The interfacial area was also modeled according to Bravo et al.^43^ We fixed the interfacial area factor to a default of 1. The heat transfer has been modeled using the Chilton and Colburn analogy. The liquid holdup was predicted using the Billet and Schultes correlation^44^. The liquid film was discretized into 6 segments to calculate the liquid mass transfer resistance, as suggested by Zhang et al.,^40^ while the vapor phase film resistance was modeled assuming no reaction takes place in the vapor phase. The reaction condition factor and transfer condition factor were set to their default, 0.5. The hydraulics were modeled assuming that the column is composed of 50 mixed stages.

**Table 2:** RadFrac Specification and Submodels Used

features	specification
calculation type	rate-based
number of stages	50
mass transfer coefficient model	Brf-85
interfacial area model	Brf-85
interfacial area factor	1
heat transfer coefficient model	Chilton and Colburn analogy
holdup correlation	Billet-93
flow model	mixed
reaction film resistance	discretized film reactions for the liquid phase, consider film for the vapor phase
reaction condition factor	0.5
transfer condition factor	0.5

The absorber validation has been carried out considering that the reactions are kinetics-controlled, while for the stripper validation, the chemistry was modeled using equilibrium reactions. This modeling approach is usually preferred for the stripper since there is a general lack of experimental kinetics data at stripper conditions^2^ and it is reasonably assumed that, at the stripper conditions, the reactions are so fast that the mass transfer is the limiting process.^45^

### Pilot Campaign at the Technology Centre of Mongstad (TCM)

Morgan^8^ reported 7 steady-state runs collected at TCM, where a blend of 3 M AMP and 1.5 M PZ is used to treat natural gas-based combined cycle turbine flue gas. The flue gas CO_2_ concentration is reported to be ∼3.5 vol %.^8^ However, it is not clear whether the concentration is defined on a wet or a dry basis. In this work, we assumed a concentration of 3.5 vol % on a dry basis since it provides a better fit between the experimental data and simulated results. We assumed that the flue gas entering the absorber column is saturated with water at the reported flue gas temperature. N_2_ is considered the only inert in the system. The temperature of the lean stream is also not reported, and we assumed it to be 40 °C for all the cases. Under these assumptions, the reported data are enough to validate the absorber section; however, data for the stripper validation, such as the inlet temperature of the rich entering the stripper or the overhead condenser temperature, are missing, and therefore, it is not possible to validate our Aspen model with the stripping section for this campaign. In the TCM campaign, the absorber packing height available for mass transfer was changed from 12 to 24 m by feeding the lean liquid to different sections of the column. The absorber’s inner diameter is 3 m^46^ and the packing used is Flexipac 2X.^8^ The reported 7 experimental runs cover a range of CO_2_ capture rates from 90 to 98%, and the liquid to flue gas ratio varies from 0.57 to 1.09 [kg/kg]. The absorber geometry and operative conditions are reported in Table 3.

**Table 3:** Absorber and Stripper Geometry and Operative Conditions during the Pilot Campaign at the University of Kaiserslautern^9^ and Absorber Geometry and Operative Conditions during the Pilot Campaign at the Technology Centre of Mongstad (TCM)^8^

**University of Kaiserslautern**	
**Absorber Geometry**	
column internal diameter [m]	0.125
packing height [m]	4.25
packing	BX 500
**Stripper Geometry**	
column internal diameter [m]	0.125
packing height [m]	2.55
packing	BX 500
**Operative Conditions**	
liquid flow rate [kg/h]	35–225
flue gas flow rate [kg/h]	40.4–80.2
CO_2_ concentration flue gas wet [vol %]	5.05–10.03
amine concentration [wt %]	28% AMP + 17% PZ
lean loading [ $\frac{{\mathrm{mol}}_{{\mathrm{CO}}_{2}}}{{\mathrm{mol}}_{\mathrm{AMP}}+{\mathrm{mol}}_{\mathrm{PZ}}}]$	0.011–0.224
rich loading [ $\frac{{\mathrm{mol}}_{{\mathrm{CO}}_{2}}}{{\mathrm{mol}}_{\mathrm{AMP}}+{\mathrm{mol}}_{\mathrm{PZ}}}]$	0.397–0.595
CO_2_ capture rate [%]	69.09–91.57
absorber pressure [bar]	0.99–1.04
stripper pressure [bar]	2.00
reboiler duty [kW]	5.82–14.48
reboiler duty heat loss [kW]	0.74–1.51
condenser temperature [°C]	14.74–18.41

**Technology Centre of Mongstad**	
**Absorber Geometry**	
column internal diameter [m]	3
packing height [m]	12–24
packing	FLEXIPAC 2X
**Operative Conditions**	
liquid flow rate [kg/h]	34872–65384
flue gas flow rate [kg/h]	59803–70675
CO_2_ concentration flue gas dry [vol %]	3.5
amine concentration [molality]	4.490–5.541 m AMP 2.314–2.667 m PZ
lean loading [ $\frac{mol_{CO_{2}}}{mol_{AMP}+mol_{PZ}}]$	0.064–0.236
rich loading [ $\frac{mol_{CO_{2}}}{mol_{AMP}+mol_{PZ}}]$	0.419–0.608
CO_2_ capture rate [%]	90.16–98.44
absorber pressure [bar]	assumed to 1 bar

### Pilot Campaign at the University of Kaiserslautern

Mangalapally et al.^47^ compared the process performances on a pilot scale of 32 mass % 1,2-ethanediamine, 30 mass % ethanolamine and a blend of 28 mass % AMP and 17 mass % PZ. The PhD dissertation by Mangalapally^9^ constitutes a very detailed work that is highly useful for process model validation. The pilot plant’s operative conditions during the campaigns using aqueous AMP and PZ are reported in Mangalapally’s PhD dissertation^9^ and have been used for the validation of the model developed in this work. The absorber and stripper geometry and the range of the operative conditions are reported in Table 3. The absorber has a packing height of 4.25 m, and the stripper has a packing height of 2.55 m. The absorber and the stripper are equipped with a structured packing BX 500 by Sulzer and have an internal diameter of 0.125 m. A washing section on top of the absorber is used to reduce solvent loss. The washing section is equipped with 0.42 m of structured packing Mellapak 250Y by Sulzer. The campaign consists of 17 experimental runs that cover a range of CO_2_ capture rates from 69% to 92%, with most of the runs aiming at a 90% CO_2_ capture rate. The liquid and flue gas ratio varies from 0.46 to 2.90 [kg/kg], and the CO_2_ concentration in the flue gas was investigated at 5.05 and 10.03% (wet basis). Table 3 summarizes the operative conditions and geometrical input of the pilot campaign.

The accuracy of the pilot data was assessed by calculating the deviation of the material balance from the overall plant. The CO_2_ capture has been calculated based on the composition and mass flow rate of the gas entering and leaving the absorber, the liquid analysis, and the mass flow of the gas leaving the stripper condenser. The amine content was calculated by gas chromatography, the CO_2_ content in the liquid was determined by the BaCl_2_ method, and the water content was determined by Karl Fischer titration. The absolute average deviation between the CO_2_ capture calculated using the gas analysis on the absorber side and the liquid analysis is 1.7%, while the absolute average deviation between the CO_2_ capture calculated using the gas analysis on the absorber side and the CO_2_ stripped on the stripper side is 0.3%. The amine mass fraction for the liquid streams was reported as the sum of AMP + PZ.^9^ Therefore, we assumed that the distribution between the AMP and PZ ratio is constant to the designed values of 28 mass % AMP and 17 mass % PZ for the whole campaign.

The absorber and stripper columns are equipped with different thermocouples to measure the temperature profile development within the columns. Additionally, liquid samples from the absorber and stripper sections are taken and analyzed for the CO_2_ content to evaluate the CO_2_ concentration profile along the columns.

## Results and Discussion

### Thermodynamic Modeling Results

The thermodynamic modeling results section is divided into three different parts. The first part describes the performance of the AMP/H_2_O/CO_2_ model developed in this work and the performance of the PZ/H_2_O/CO_2_ model developed by Aspen Tech on the CO_2_ solubility and liquid speciation. The second part describes the performance of the AMP/PZ/H_2_O NRTL model developed in this work, and finally, the third part describes the performance of the AMP/PZ/H_2_O/CO_2_ model developed in this work.

### AMP/H_2_O/CO_2_ and PZ/H_2_O/CO_2_

The experimental data used in the fitting of the AMP/H_2_O and AMP/H_2_O/CO_2_ system are reported together with the associated average absolute relative deviation (AARD) in Table 4. The Flash Block in Aspen Plus was used to obtain the equilibrium CO_2_ and amine partial pressure for the solutions studied in this work. The AMP/H_2_O model predicts the AMP volatility within 5.5% AARD and the solvent pressure within 3.9% AARD; the liquid heat capacity and excess enthalpy of mixing for AMP/H_2_O are also well predicted with an AARD of 1.9% and 6.3%, respectively. Figure 1 shows that the model accurately predicts the amine volatility for the AMP/H_2_O on the data set by Hartono et al.^17^ When compared to the data by Mathonat et al.,^23^ the model slightly underestimates the excess enthalpy of mixing of AMP/H_2_O in the region where the highest heat is developed. The NRTL model developed in this work shows good predictions of both the VLE and calorimetric data of AMP/H_2_O solutions.

**Table 4:** Dataset Used in the Regression of the AMP/H_2_O/CO_2_ System Together with the AARD Values

**AMP/H**_**2**_ **O**						
**data type**	**CO**_**2**_ **loading [** $\frac{\mathrm{mo}l_{{\mathrm{CO}}_{2}}}{\mathrm{mo}l_{\mathrm{AMP}}}]$	**temperature [°C]**	**amine concentration**	**data points**	**AARD [%]**	**reference**
*PTx*		10.3–89.8	2.5 M	7	1.9 *on P_TOT_*	Nouacer et al.^18^
*PTxy*		60–100	x_AMP_ = 0.023 – 0.30	26	0.8 *on P*_TOT_ 6.7 *on y*_*AMP*_	Hartono et al.^17^
*PTx*		20–100	x_AMP_ = 0.05 – 0.82	64	9.1 *on P_TOT_*	Belabbaci et al.^16^
*PTxy*		91–164	x_AMP_ = 0.12 – 0.90	21	0.1 *on T* 4.2 *on y*_*AMP*_	Pappa et al.^19^
**Average Error** *P_TOT_* **[%]**					**3.9**	
**Average Error** *y*_*AMP*_ **[%]**					**5.5**	
*c*_*P*_		5–95	x_AMP_ = 0.06 – 1	211	2.3	Zhang et al.^22^
*c*_*P*_		30–80	x_AMP_ = 0.2 – 0.8	44	2.2	Chen et al.^52^
*c*_*P*_		30–80	x_AMP_ = 0.2 – 1	55	1.3	Chiu et al.^21^
**Average Error** *c*_*P*_ **[%]**					**1.9**	
*H* ^*E*^		35	x_AMP_ = 0.05 – 0.9	16	6.3	Mathonat et al.^23^
**Average Error** *H* ^*E*^ **[%]**					**6.3**	

**AMP/H**_**2**_ **O/CO**_**2**_						
**data type**	**CO**_**2**_ **loading [** $\frac{\mathrm{mo}l_{CO_{2}}}{\mathrm{mo}l_{\mathrm{AMP}}}]$	**temperature [°C]**	**amine concentration**	**data points**	**AARD [%]**	**reference**
*P*_*CO*_2__	0–1.04	40–80	0.1,1,3 M	109	20.7	Hartono et al.^24^
*P*_*CO*_2__	0.40–0.87	40–100	wt_AMP_ = 0.30	28	25.3	Li et al.^25^
*P*_*CO*_2__	0–1.10	25–55	2.5,3.4,4.9 M	166	21.7	Dash et al.^26^
*P*_*CO*_2__	0.41–0.97	30–50	2,2.8,3.4 M	51	15.3	Kundu et al.^27^
**Average Error** *P*_***CO*_2_**_ **[%]**					**20.7**	
*P*_*TOT*_	0.06–1.01	40–80	3 M	50	12.1	Morlando et al.^4^
*P*_*TOT*_	0.07–0.96	40–120	3 M	65	10.0	Hartono et al.^12^
*P*_*TOT*_	0–1.95	100–120	0.1,1,3 M	53	12.9	Hartono et al.^24^
*P*_*TOT*_	0–0.92	40–120	wt_AMP_ = 0.30	45	8.4	Tong et al.^28^
**Average Error** *P*_*TOT*_ **[%]**					**10.4**	
Δ*H*_*abs*_	0.06–1.01	40–80	3 M	50	11.5	Morlando et al.^4^
**Average Error** Δ*H*_*abs*_ **[%]**					**11.5**	

**Figure 1:**
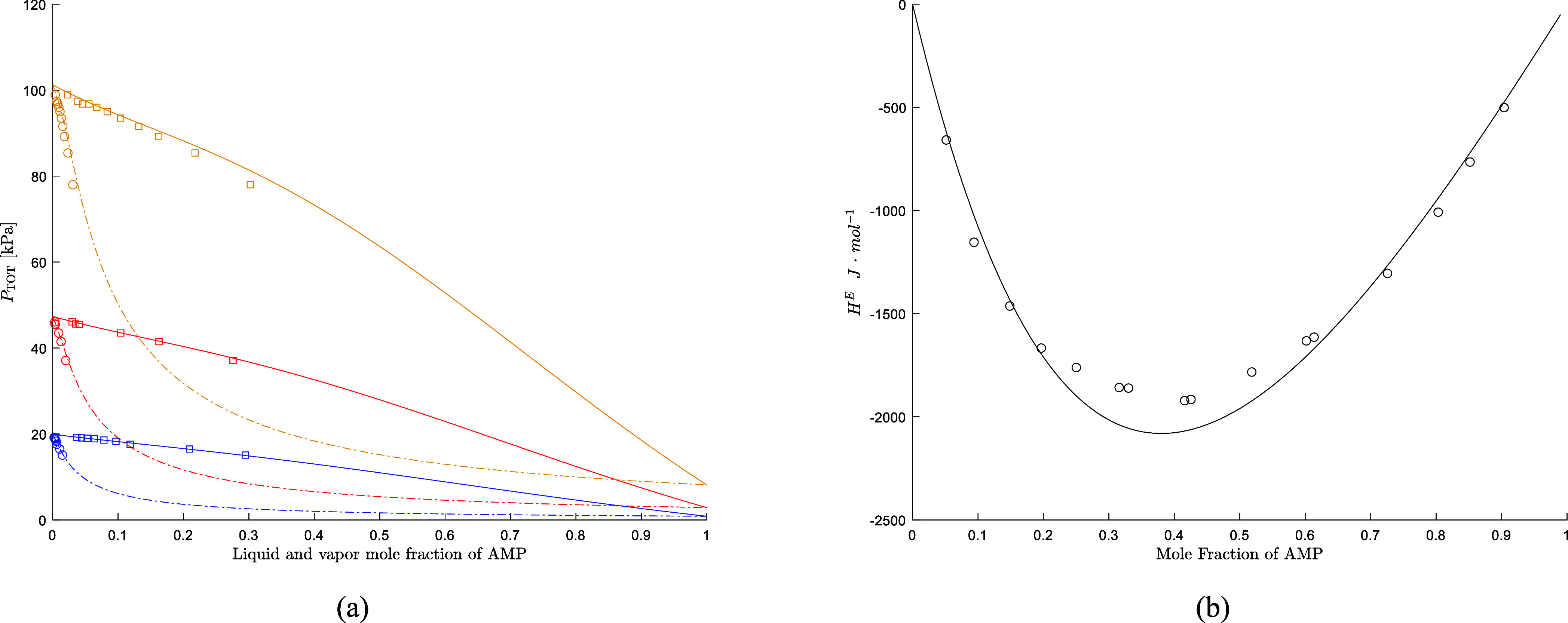
(a) Vapor liquid equilibrium prediction for AMP/H_2_O (box open, liquid mole fraction; circle open, vapor mole fraction Hartono et al.,^17^ – equilibrium liquid concentration, −. – equilibrium vapor concentration) (blue 60 °C, red 80 °C, yellow 100 °C). (b) Excess enthalpy prediction for AMP/H_2_O at 35 °C, (circle open, Mathonat et al.^23^).

The fitting data sets for the AMP/H_2_O/CO_2_ cover a range of amine concentration from 0.1 to 4.9 M, temperature from 25 to 120 °C and CO_2_ loading from 0 to 1.95 $\frac{{\mathrm{mol}}_{{\mathrm{CO}}_{2}}}{{\mathrm{mol}}_{\mathrm{AMP}}}$ . The model represents the data well with an AARD of 20.7% for the CO_2_ partial pressure, *P*_*CO*_2__, and 10.4% for the total pressure, *P*_*tot*_. The model has been tested on the recent data collected by François et al.^48^ at a lower amine concentration than the fitting range, i.e. 0.06 M. The model predicts the data well, Figure S1, indicating that the model can be reasonably extrapolated at lower concentrations. However, as usual, one should always check the predictions of the model when it is used outside the fitting range. Figure 2 shows the model prediction of the CO_2_ solubility over an aqueous solution of 3 M AMP, which is the relevant concentration for the CESAR1 blend. The data by Morlando et al.,^4^ Hartono et al.,^24^ Tontiwachwuthikul et al.,^49^ Yang et al.,^50^ and Roberts and Mather^51^ are used for the validation. The partial pressure of CO_2_ data of Hartono et al.^24^ at 100 and 120 °C were estimated by the difference between the total pressure at a specific loading and the pressure of the unloaded solvent, similarly as it has been done by Morlando et al.^4^ The model predicts the data well from 40 to 120 °C, which is the conventional operative range for the CO_2_ absorption process. Additionally, the model predicts well the liquid speciation data by Ciftja et al.^29^ as shown in Figure 2.

**Figure 2:**
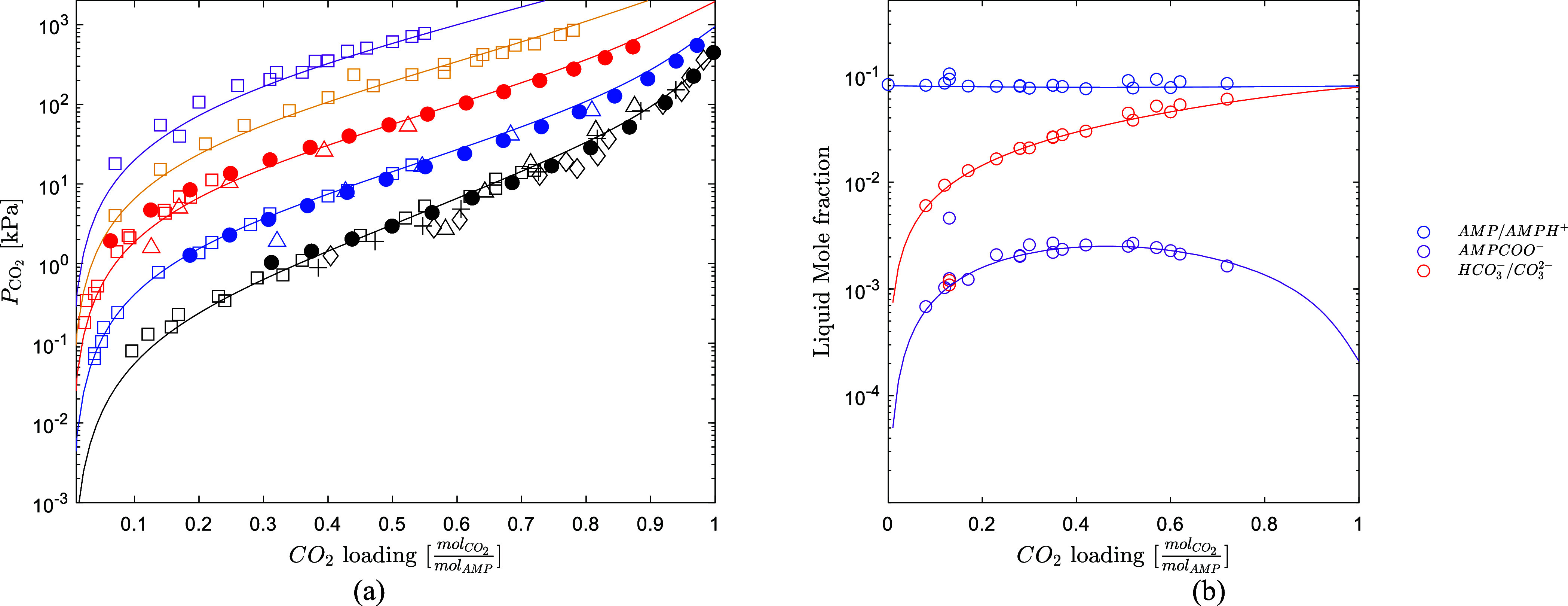
(a) CO_2_ solubility prediction over an aqueous 3 M AMP solution, (solid circle, Morlando et al.,^4^ square open, Hartono et al.,^24^ triangle up open, *T*ontiwachwuthikul et al.,^49^ +, Yang et al.,^50^ diamond open, Roberts et al.^51^) (black 40 °C, blue 60 °C, red 80 °C, yellow 100 °C, purple 120 °C). (b) Liquid speciation at 25 °C, (circle open, Ciftja et al.^29^).

The predictions for the heat of absorption of CO_2_ in an aqueous solution of 3 M AMP are shown in Figure 3. The differential heat of absorption of CO_2_ has been calculated by energy balance using the Flash Block in Aspen Plus, similarly, as described by Zhang et al.^6^ The model predicts that the heat of absorption decreases as the temperature increases, while the experimental data indicate that the heat of absorption is rather constant in the temperature range investigated. The model correctly predicts the rapid decrease as a function of the CO_2_ loading. The model predictions are very good at 40 and 60 °C but worse at 80 °C. Overall, the model gives a good representation of the heat of absorption with an AARD of 11.5% on the fitting data set.

**Figure 3:**
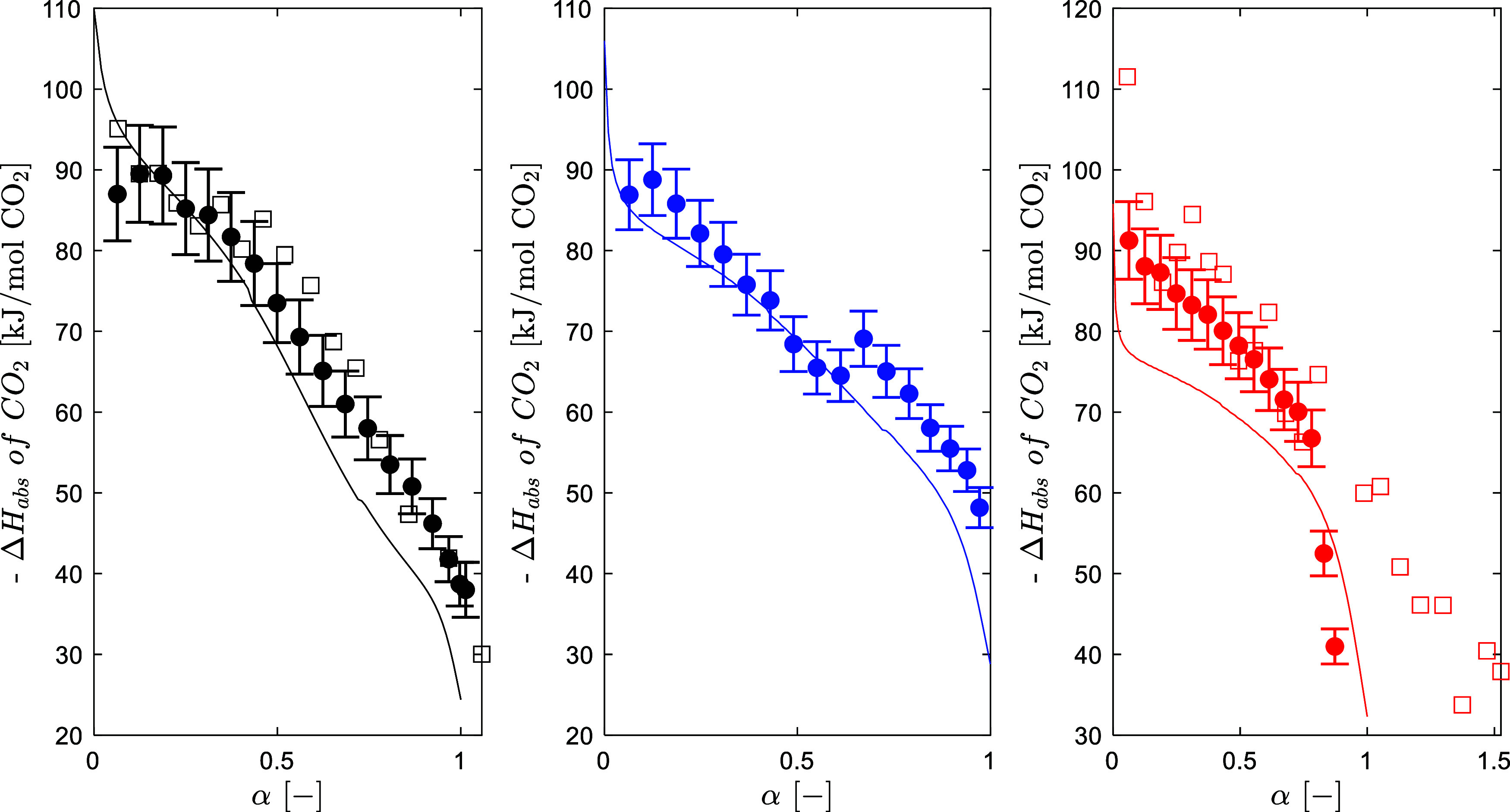
Heat of absorption of CO_2_ in aqueous 3 M AMP solution (circle solid, Morlando et al.,^4^ square open, Kim^53^) (black 40 °C; blue, 60 °C; red, 80 °C).

The model available in Aspen Plus was used in this work to characterize the thermodynamics of the PZ/H_2_O/CO_2_ system. Figure 4 shows that the model available in Aspen Plus accurately predicts well the CO_2_ solubility data by Ermatchkov et al.^54^ at 1.7 M PZ from 40 °C up to 120 °C. Figure 4 also shows that the model predicts well the liquid speciation data by Ermatchkov et al.,^55^ indicating a good prediction of the chemical equilibria of the system. Based on this validation, the thermodynamic framework for PZ/H_2_O/CO_2_ from AspenTech can be used as such to model the quaternary system.

**Figure 4:**
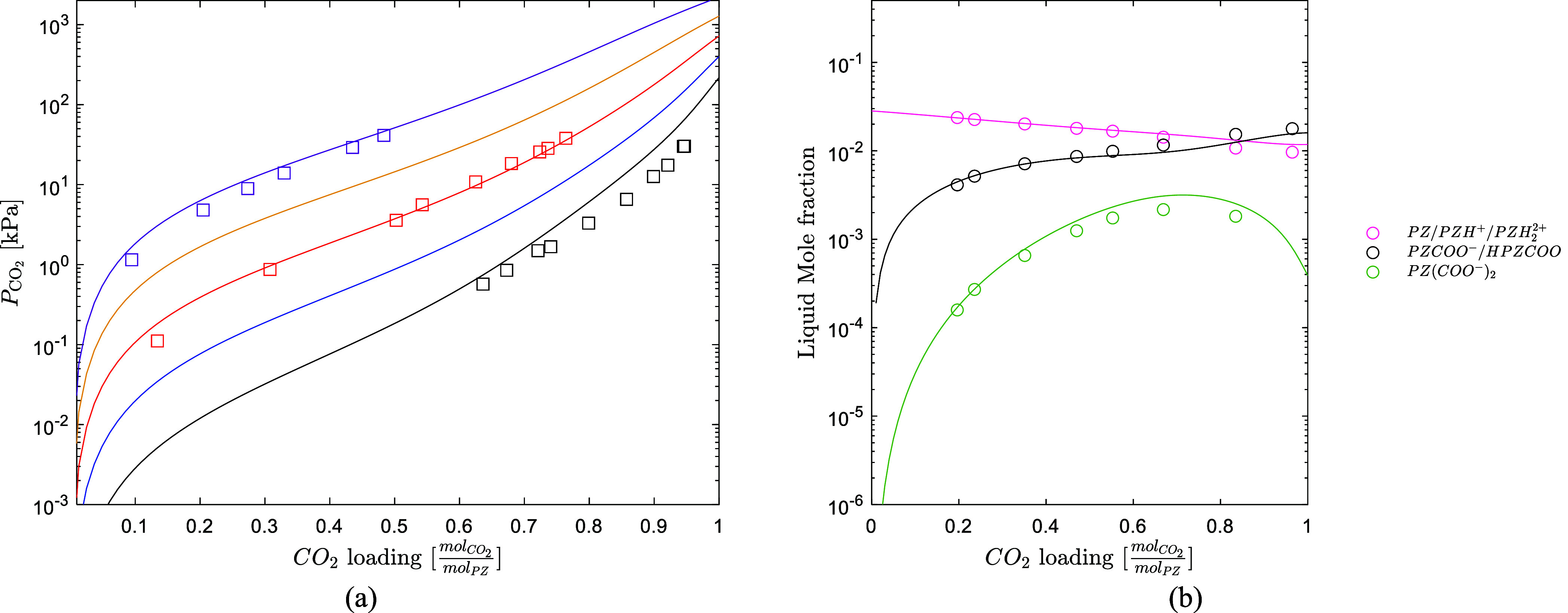
(a) CO_2_ solubility prediction over an aqueous 1.7 M PZ solution, Ermatchkov et al.^54^ (black 40 °C, blue 60 °C, red 80 °C, yellow 100 °C, purple 120 °C). (b) Speciation prediction over an aqueous 11.1 mass % PZ solution at 25 °C (circle open, Ermatchkov et al.^55^).

### AMP/PZ/H_2_O

The models described above were used to develop an e-NRTL framework for ternary and quaternary systems AMP/PZ/H_2_O and AMP/PZ/H_2_O/CO_2_. As previously discussed, the model accuracy of the amine volatility was tested on the data by Hartono et al.^17^ and the recent data published by Charalambous et al.,^30^ while the model accuracy of the liquid heat capacity was tested on the data by Chen et al.^20^. The data set by Hartono et al.^17^ covers temperatures from 60 to 100 °C, while the data by Charalambous et al.,^30^ which is focused on the characterization of the water wash section, covers temperatures from 30 to 50 °C and extends the range of concentration of AMP and PZ previously investigated. The model predicts the total pressure very well, with an AARD of 0.5% and 1.8% for the measurements by Hartono et al.^17^ and Charalambous et al.,^30^ respectively. The partial pressures of AMP and PZ are predicted with an AARD of 19.3% and 42.4%, respectively, on the Hartono et al. data set and with an AARD of 40.4% and 80.6%, respectively, on the Charalambous et al. data set. Figure 5 shows that the model does not present any significant trend as a function of the temperature, and the high AARD for the data sets can be explained by the experimental uncertainty and scattering of the data. The AARDs of the partial pressure of AMP and PZ for the Charalambous et al.^30^ data set are around two times higher than for the Hartono et al.^17^ data. At lower temperatures, the equilibrium amine vapor concentrations are lower, reaching the ppm level for piperazine, resulting in more challenging measurements. The model predicts the heat capacity for aqueous AMP/PZ solutions data by Chen et al.^20^ within an AARD of 2.6%, which is considered satisfactory.

**Figure 5:**
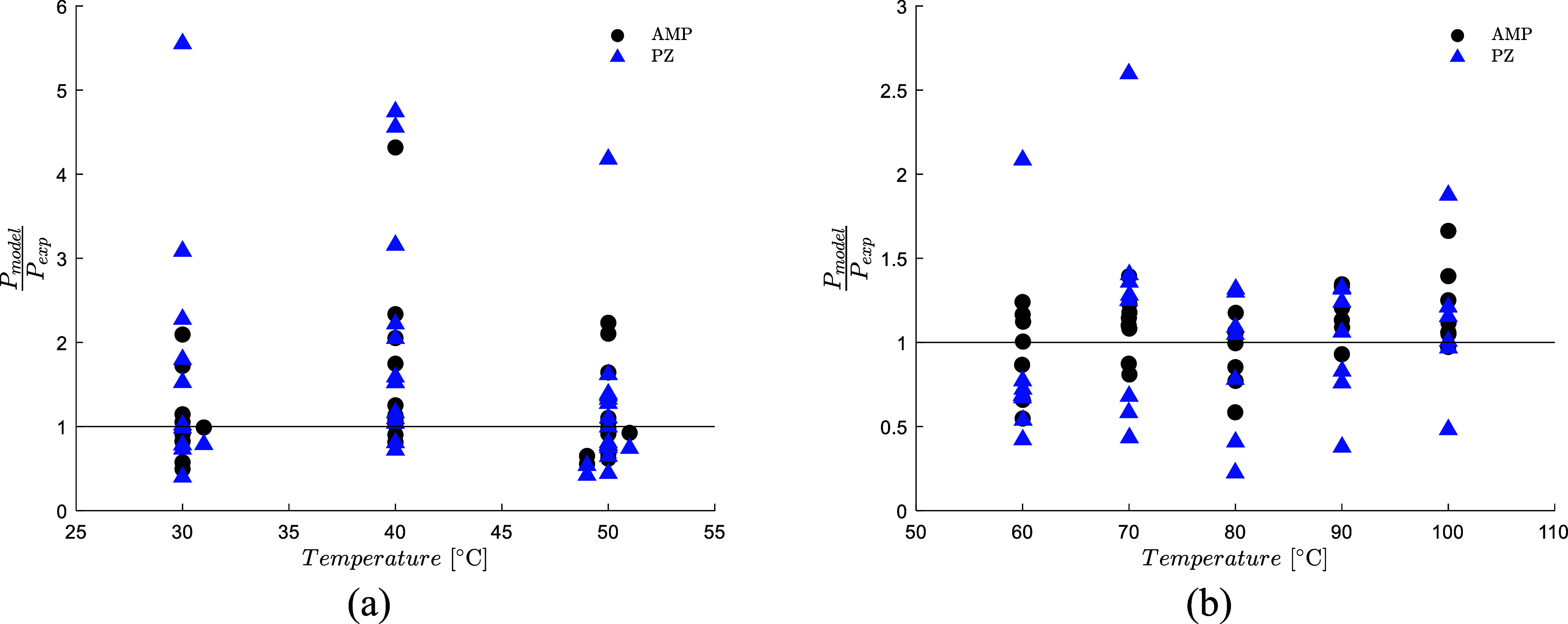
(a) Model prediction of AMP and PZ partial pressure on the Charalambous et al.^30^ data set, (b) Model prediction of AMP and PZ partial pressure on the Hartono et al.^17^ data set.

### AMP/PZ/H_2_O/CO_2_

The experimental data used in the fitting of the AMP/PZ/H_2_O/CO_2_ system are reported together with the associated average absolute relative deviation (AARD) in Table 5.

**Table 5:** Data Sets Used in the Regression of AMP/PZ/H_2_O/CO_2_ System Together with AARD Values

**data type**	**CO_2_** **loading** $\left[\frac{{\mathrm{mol}}_{{\mathrm{CO}}_{2}}}{{\mathrm{mol}}_{\mathrm{AMP}}+{\mathrm{mol}}_{\mathrm{PZ}}}\right]$	**temperature [°C]**	**amine concentration**	**data points**	**AARD [%]**	**reference**
*P*_CO_2__	0.035–0.73	40–80	3.0 M AMP+1.5 M PZ	43	14.6	Hartono et al.^12^
*P*_CO_2__	0.36–0.83	20–160	[m_AMP_/m_pz_] = 2.3/5,4/2 m	78	17.4	Li et al.^31^
*P*_CO_2__	0.218–1.029	30–55	wt_AMP_ = 0.32 to 0.48, wt_PZ_ = 0.02 to 0.08	175	39.2	Dash et al.^32^
*P*_CO_2__	0.04–0.83	40–120	3.0 M AMP + 1.5 M PZ	49	25.0	Brúder et al.^34^
*P*_CO_2__	0.20–1.07	25–55	wt_AMP_ = 0.22 to 0.28, wt_PZ_ = 0.02 to 0.08	158	35.0	Dash et al.^33^
average error *P*_CO_2__[%]					26.2	
*P*_TOT_	0.03–0.94	40–150	wt_AMP_ = 0.244 to 0.350, wt_PZ_ = 0.050 to 0.157	274	7.0	Morlando et al.^4^
average error *P*_TOT_ [%]					7.0	
species concentration	0–0.78	25	3.0 M AMP + 1.5 M PZ, 1.5 M AMP + 0.75 M PZ	81	-	Morlando et al.^4^
Δ*H*_abs_	0.04–0.94	40–80	wt_AMP_ = 0.244 to 0.350, wt_PZ_ = 0.050 to 0.157	129	10.2	Morlando et al.^4^
average error Δ*H*_abs_ [%]					10.2	
*x*_CO_2__	0.11–0.52	25–80	3.0 M AMP + 1.5 M PZ		13.1	Morlando et al.^4^
average error *x*_CO_2__ [%]					13.1	

The fitting data sets cover a range of AMP concentration from 12 to 48 mass % and PZ concentration from 2 to 26 mass %, temperature from 20 to 160 °C, and CO_2_ loading from 0 to 1.03. The CO_2_ solubility, as partial pressure of CO_2_, over aqueous AMP/PZ solutions is predicted to be within an AARD of 26.3%. The AARD error for the CO_2_ partial pressure is in line with other models, built for different amine systems, using data sets from different sources.^11,12,56^ The model predicts the CO_2_ solubility, as total pressure, over aqueous AMP/PZ solutions within an AARD of 7.0%.

Figure 6 shows the model prediction of the CO_2_ solubility over the CESAR1 blend from 40 to 150 °C. The model slightly underpredicts the CO_2_ partial pressure at 80 °C for loading higher than 0.60 $\frac{{\mathrm{mol}}_{{\mathrm{CO}}_{2}}}{{\mathrm{mol}}_{\mathrm{AMP}}+{\mathrm{mol}}_{\mathrm{PZ}}}$ but provides overall a great representation of the data. François et al.^48^ measured CO_2_ solubility data over 10 and 50 times diluted CESAR1 solutions. The data were not used in the model fitting, but the model still represents the data well. Figure 6 shows the comparison of the predicted CO_2_ solubility over a solution of 0.3 M AMP + 0.15 M PZ. This indicates that the model can give a good prediction of the CO_2_ solubility in aqueous AMP/PZ solutions also at water wash relevant operative conditions.

**Figure 6:**
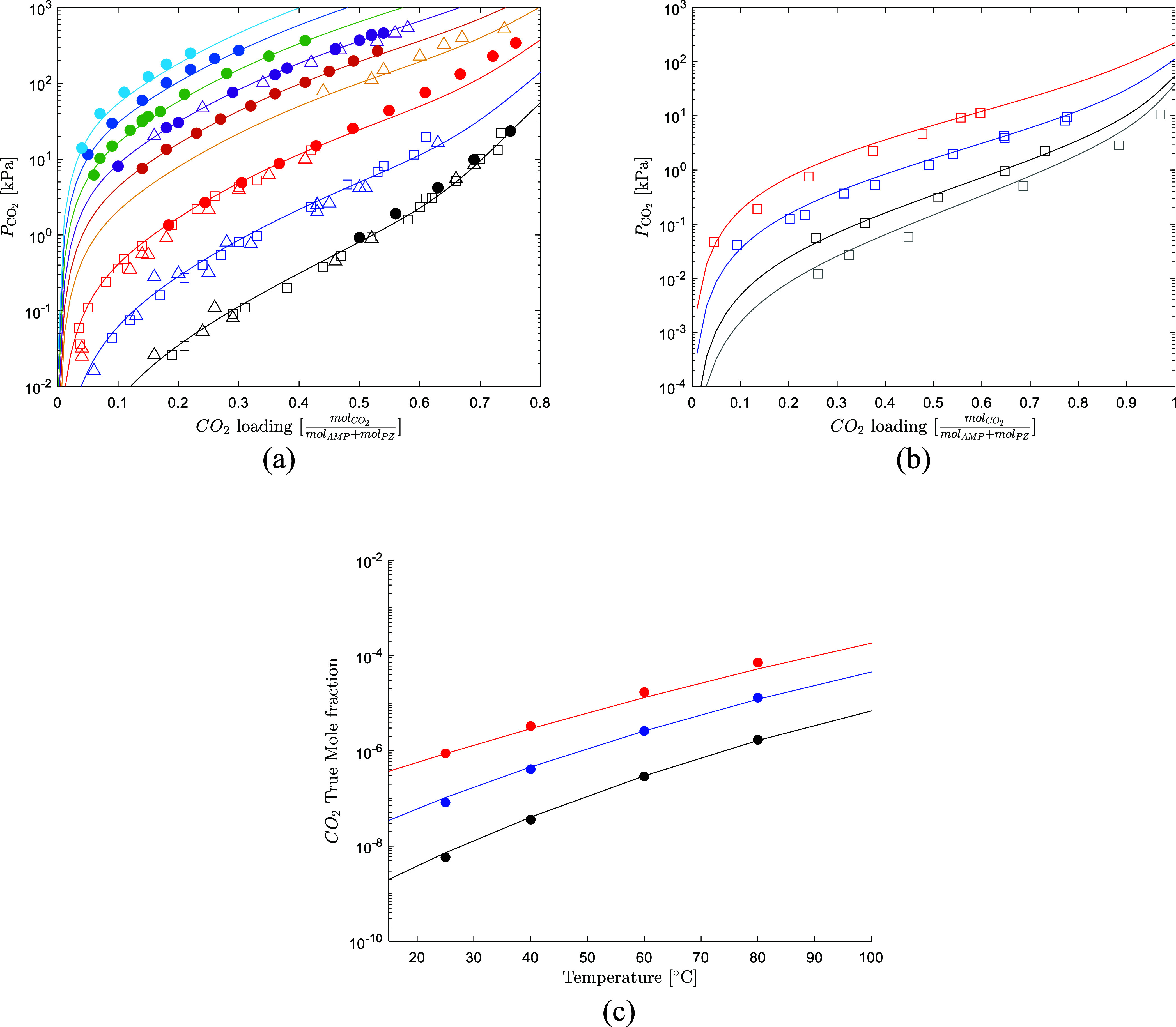
(a) CO_2_ solubility of the CESAR1 blend (circle solid, Morlando et al.,^4^ square open, Hartono et al.,^12^ triangle up solid, Brúder et al.^34^) (black 40 °C, blue 60 °C, red 80 °C, yellow 100 °C, orange 110 °C, purple 120 °C, green 130 °C, dark blue 140 °C, cyan 150 °C). (b) CO_2_ solubility of an aqueous solution of 0.3 M AMP + 0.15 M PZ (square open, François et al.^48^) (gray 30 °C, black 40 °C, blue 60 °C, red 80 °C). (c) CO_2_ true mole fraction as a function of the system temperature (circle solid, Morlando et al.^4^) (black 0.11 $\frac{\mathrm{mo}l_{CO_{2}}}{\mathrm{mo}l_{\mathrm{AMP}}+\mathrm{mo}l_{\mathrm{PZ}}}$ , blue 0.29 $\frac{\mathrm{mo}l_{CO_{2}}}{\mathrm{mo}l_{\mathrm{AMP}}+\mathrm{mo}l_{\mathrm{PZ}}}$ , red 0.52 $\frac{\mathrm{mo}l_{CO_{2}}}{\mathrm{mo}l_{\mathrm{AMP}}+\mathrm{mo}l_{\mathrm{PZ}}}$ ).

The free concentration of CO_2_ in the mixed solvent has been estimated from the N_2_O solubility data by Morlando et al.^4^ The N_2_O Henry’s constant has been treated using the N_2_O analogy to obtain the CO_2_ apparent Henry’s constant for the CO_2_ loaded CESAR1 solution. The mole fraction of the free CO_2_ has been estimated as shown in eq 14. The partial pressure of CO_2_ has been calculated by the model, while the H_CO_2__^exp^ has been obtained through the N_2_O analogy.

$$x_{{\mathrm{CO}}_{2}}^{\mathrm{free},\exp}=\frac{P_{{\mathrm{CO}}_{2}}^{\mathrm{mod}}}{H_{{\mathrm{CO}}_{2}}^{\exp}}$$ 14

Figure 6 shows the model predictions for the true mole fraction of the CO_2_. The model predicts well the free CO_2_ concentration in the CO_2_ loading range, 0.11 to 0.52 $\frac{{\mathrm{mol}}_{{\mathrm{CO}}_{2}}}{{\mathrm{mol}}_{\mathrm{AMP}}+{\mathrm{mol}}_{\mathrm{PZ}}}$ , and temperature range, 25 to 80 °C, with an AARD of 13.1%.

The model accurately predicts the liquid speciation of the solvent, as shown in Figure 7. Only the data set from Morlando et al.^4^ was used in the regression. The model accurately predicts the speciation data at the CESAR1 concentration and a lower concentration (1.5 M AMP + 0.75 M PZ) in a CO_2_ loading range from 0 to 0.78 $\frac{{\mathrm{mol}}_{{\mathrm{CO}}_{2}}}{{\mathrm{mol}}_{\mathrm{AMP}}+{\mathrm{mol}}_{\mathrm{PZ}}}$ . Li et al.^10^ also generated some NMR data for aqueous AMP/PZ solutions. The NMR data by Li et al. were not used in the regression, but the model predicts still reasonably well the data, as shown in Figure S2.

**Figure 7:**
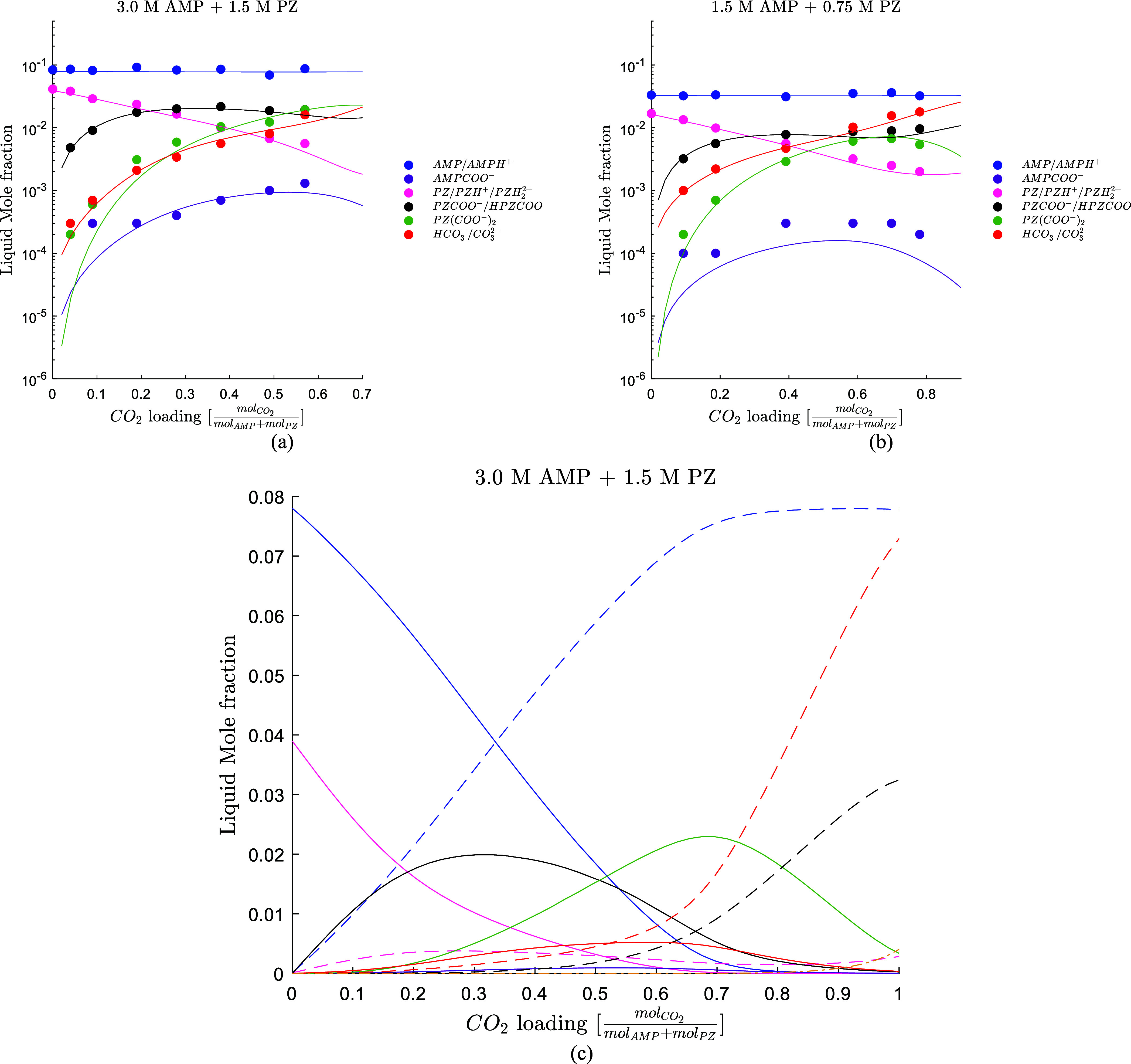
(a) Model predictions for the speciation of the CESAR1. (b) Model prediction for the speciation of 1.5 M AMP + 0.75 M PZ. Data from Morlando et al.^4^ (blue line AMP/AMPH^+^, purple line AMPCOO^–^, pink line PZ/PZH^+^, black line PZCOO^–^/HPZCOO, green line PZ­(COO^–^)_2_, red line CO_3_^2–^/HCO_3_^–^). (c) Full speciation for the CESAR1 blend at 25 °C, (blue line AMP, blue broken line AMPH^+^, purple line AMPCOO^–^, pink line PZ, pink broken line PZH^+^, black line PZCOO^–^, black broken line HPZCOO, green line PZ­(COO^–^)_2_, red line CO_3_^2–^, red broken line HCO_3_^–^, yellow broken line CO_2_).

Figure 7 also shows the predicted full speciation of the CESAR1 blend. The model indicates that the CO_2_ exists mainly as PZCOO^–^ at lower CO_2_ loadings (up to 0.50 $\frac{{\mathrm{mol}}_{{\mathrm{CO}}_{2}}}{{\mathrm{mol}}_{\mathrm{AMP}}+{\mathrm{mol}}_{\mathrm{PZ}}}$ ) while as PZ­(COO^–^)_2_ and HCO_3_^–^ at higher CO_2_ loadings. The PZCOO^–^ reaches a maximum around 0.30 $\frac{{\mathrm{mol}}_{{\mathrm{CO}}_{2}}}{{\mathrm{mol}}_{\mathrm{AMP}}+{\mathrm{mol}}_{\mathrm{PZ}}}$ , promoting the carbamation reaction to PZ­(COO^–^)_2_, whose concentration reaches a maximum at around 0.70 $\frac{{\mathrm{mol}}_{{\mathrm{CO}}_{2}}}{{\mathrm{mol}}_{\mathrm{AMP}}+{\mathrm{mol}}_{\mathrm{PZ}}}$ . As expected, given the high concentration of AMP in the solvent, HCO_3_^–^ is the major product at a high loading. CO_3_^2–^, PZH^+^, and AMPCOO^–^ are not important products in the whole CO_2_ loading range.

The heat of absorption of CO_2_ data from Morlando et al.^4^ were included in the parameters’ regression. The model predicts the calorimetric data with an AARD of 10.2%, which is considered to be satisfactory. The regression of the heat of absorption for a mixed solvent is more challenging, since only the binary energy parameters can be used in the model fitting. Figure 8 shows the predicted heat of absorption of CO_2_ compared to the data by Morlando et al.^4^ and Hartono et al.^12^ The model slightly overestimates the heat of absorption compared to Hartono et al.^12^ and underestimates the data from Morlando et al.;^4^ the heat of absorption has an overall decreasing trend as a function of the CO_2_ loading.

**Figure 8:**
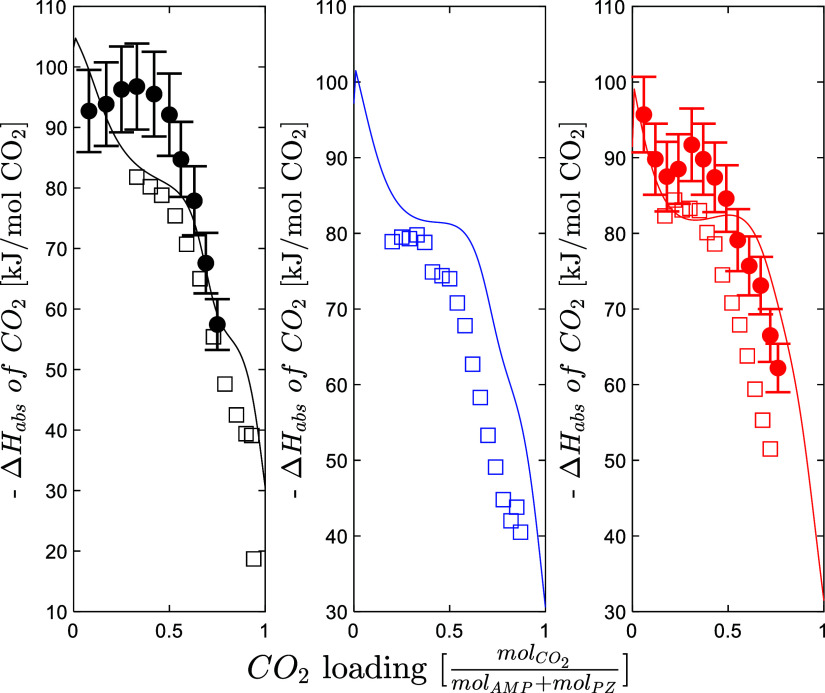
Heat of absorption of CO_2_ for the CESAR1 solvent (circle solid, Morlando et al.,^4^ square open, Hartono et al.^12^) (black 40 °C, blue 60 °C, red 80 °C).

To conclude, the thermodynamic model developed represents the CO_2_ solubility, liquid speciation, and heat of absorption of CO_2_ and CO_2_ physical solubility data of aqueous AMP/PZ solutions with good accuracy and can be used for process modeling and simulation of the CO_2_ capture process with the CESAR1 solvent.

### Process Modeling Results

The process modeling section is divided into three different sections. The first section describes the validation results of the absorber unit for the experimental campaign at the University of Kaiserslautern. The second section describes the results of the stripper unit for the experimental campaign at the University of Kaiserslautern. Finally, the third part describes the validation results of the absorber unit for the experimental campaign at the TCM.

### University of Kaiserslautern: Absorber Validation

The experimental results of CO_2_ loading in the rich stream leaving the absorber, the experimental CO_2_ capture, and the simulated results for all 17 runs at the University of Kaiserslautern are reported in Table 6. The agreement between the data and the model is very good. The model predicts the rich loading and the CO_2_ removal within an absolute average relative deviation (AARD) of 1.8% and 2.0%, respectively.

**Table 6:** Absorber Simulation Result (CO_2_ Rich Loading and CO_2_ Removal) for Validation of the Kaiserslautern Pilot Plant Data^a^ ^,^ ^9^

	**rich loading** $\left[\frac{\mathrm{mo}l_{CO_{2}}}{\mathrm{mo}l_{\mathrm{AMP}}+\mathrm{mo}l_{\mathrm{PZ}}}\right]$			CO_2_ removal [%]		
run	experimental	simulation	absolute relative deviation [%]	experimental	simulation	absolute relative deviation [%]
G1	0.562	0.558	0.8	69.06	67.48	1.6
G2	0.561	0.541	3.5	89.35	83.08	6.3
G3	0.540	0.546	1.1	91.23	90.70	0.5
G4	0.516	0.517	0.2	90.00	89.93	0.1
G5	0.484	0.481	0.5	90.30	92.26	2.0
G6	0.460	0.471	2.4	90.21	91.18	1.0
G7	0.400	0.402	0.5	90.49	91.25	0.8
G8	0.558	0.602	7.9	80.66	83.70	3.0
G9	0.558	0.582	4.3	88.68	93.07	4.4
G10	0.595	0.598	0.4	89.15	86.42	2.7
G11	0.521	0.550	5.6	90.51	95.34	4.8
G12	0.516	0.526	1.9	91.19	92.85	1.7
G13	0.480	0.481	0.2	89.33	90.29	1.0
G14	0.436	0.430	1.4	90.26	91.72	1.5
G15	0.493	0.493	0.1	91.57	91.32	0.3
G16	0.464	0.466	0.4	90.98	92.54	1.6
G17	0.480	0.481	0.2	89.33	90.32	1.0

a The absolute relative deviation is calculated as $\frac{\left|y_{\mathrm{mod}\mathrm{el}}-y_{\exp\mathrm{erimental}}\right|}{y_{\exp\mathrm{erimental}}}\times 100$

Figure 9 shows the predicted temperature and liquid CO_2_ concentration profiles for three selected cases with different CO_2_ flue gas concentrations and liquid–gas mass ratios. The model gives a good representation of the temperature and liquid CO_2_ concentration profile along the absorber column. The ratio between the predicted and experimental CO_2_ capture rate for the different cases has been plotted against the experimental rich loading, experimental lean loading, and liquid-to-gas ratio to investigate the presence of systematic error trends, Figure 10. The model demonstrates no systematic bias under the operative conditions investigated, with the CO_2_ capture deviating by less than 5% in most cases. The predicted temperature and CO_2_ concentration profiles in the absorber for the other cases are available in the Supporting Information, Figure S4.

**Figure 9:**
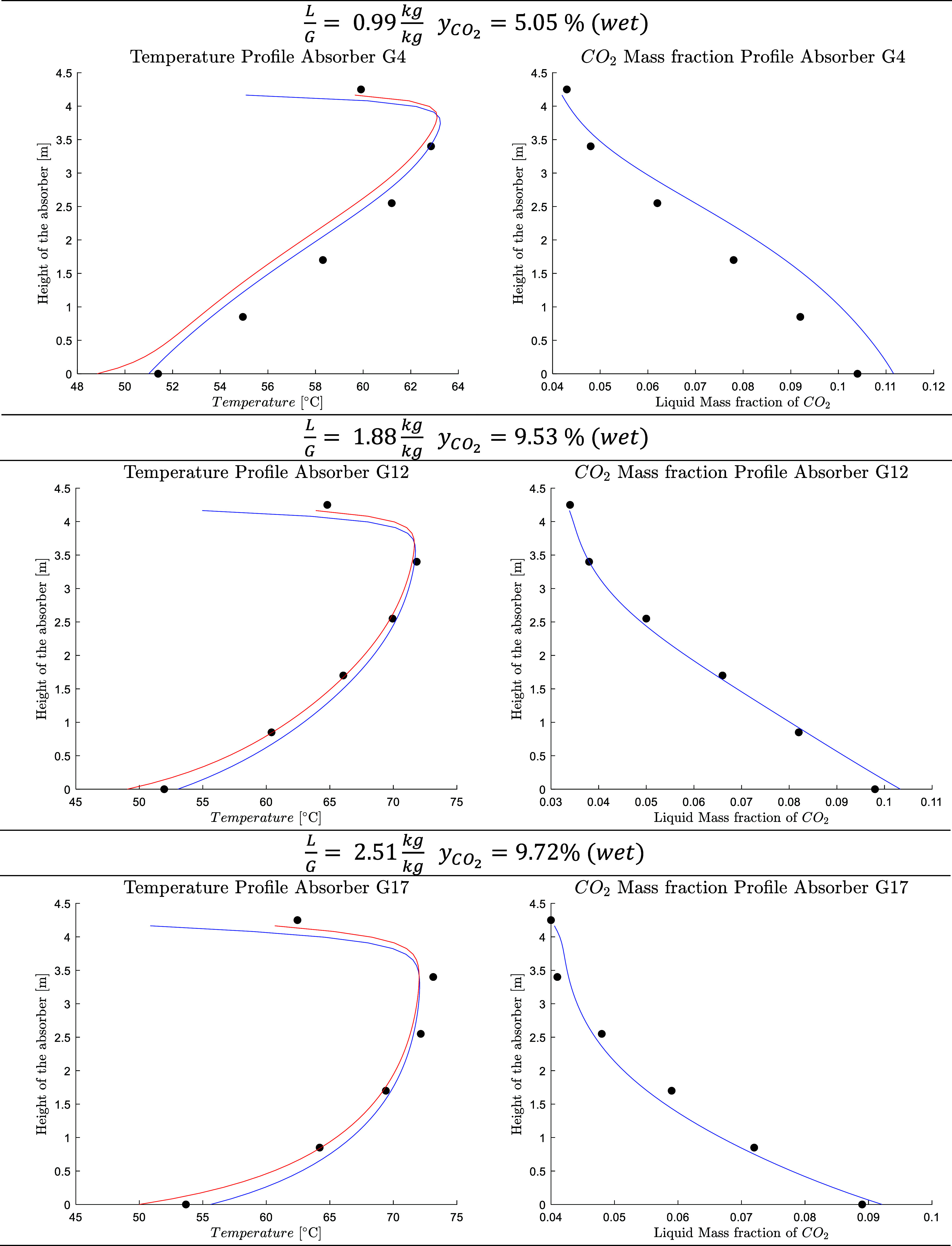
Temperature (blue line, liquid temperature; red line, vapor temperature; circle solid, experimental data) and liquid CO_2_ concentration profile along the absorber for three selected cases from Kaiserslautern.^9^

**Figure 10:**
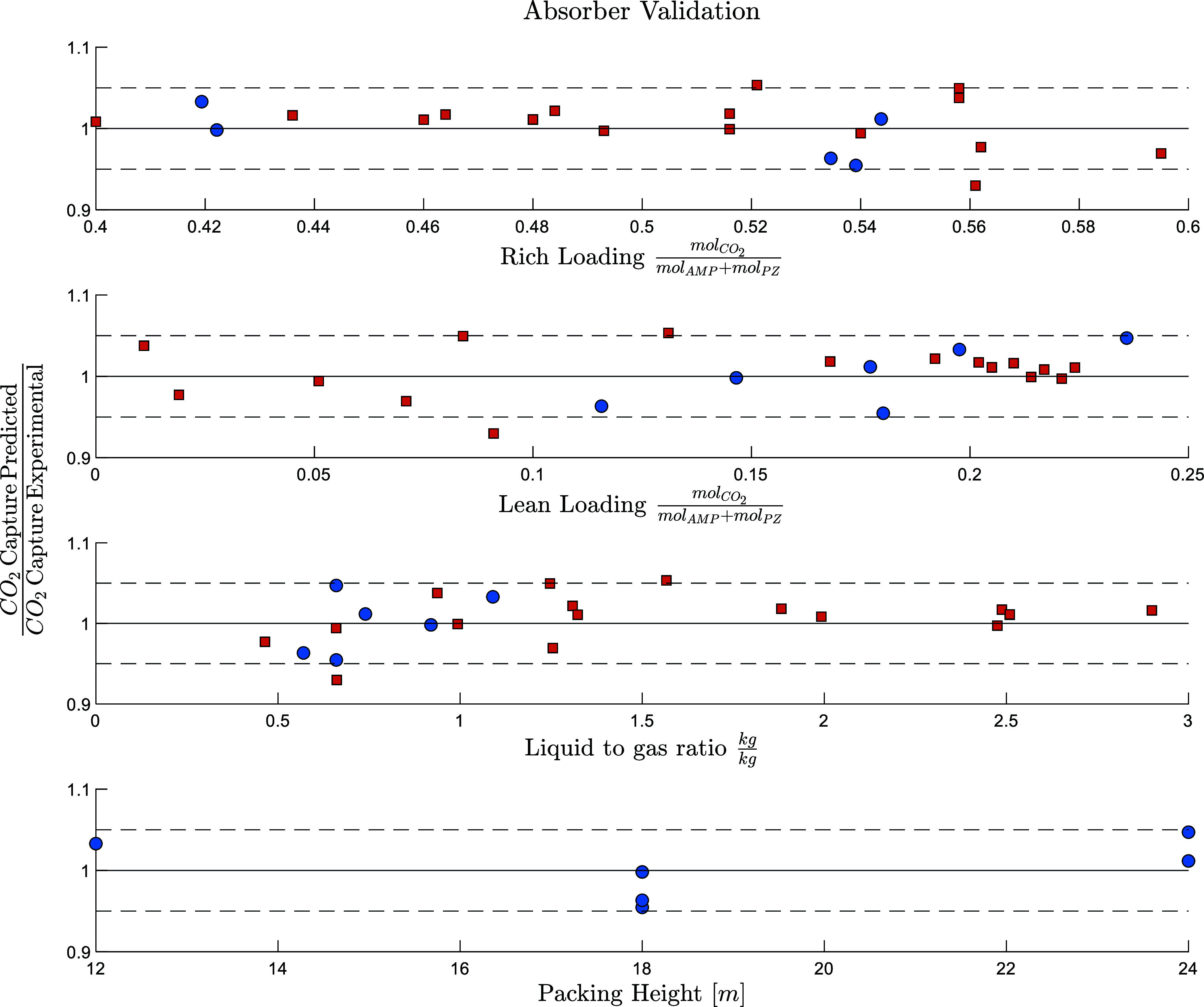
Error distribution for the absorber validation for the TCM^8^ (blue circle) and Kaiserslautern^9^ (orange squares) campaigns as a function of experimental rich loading, experimental lean loading, liquid-to-gas ratio, and packing height. Case A5 deviation was omitted in this plot.

### University of Kaiserslautern: Stripper Validation

The experimental results of CO_2_ loading in the lean stream leaving the stripper, the experimental specific reboiler duty (SRD), and the simulated results for all 17 runs are reported in Table 7. The simulation and experimental results reported in Table 7 consider heat losses on the stripping side. Mangalapally^9^ reported the heat loss for each case, which is used in the simulation. The model predicts the lean loading and the specific reboiler duty (SRD) within an absolute average relative error (AARD) of 16.0% and 4.8% respectively. The AARD of the lean CO_2_ loading is relatively high because of the low CO_2_ content in the lean stream. The absolute average deviation (AAD) on the lean loading is 0.01 $\frac{{\mathrm{mol}}_{{\mathrm{CO}}_{2}}}{{\mathrm{mol}}_{\mathrm{AMP}}+{\mathrm{mol}}_{\mathrm{PZ}}}$ and it is considered satisfactory.

**Table 7:** Stripper Simulation Result (CO_2_ Lean Loading and Specific Reboiler Duty) for Validation of the Kaiserslautern Pilot Plant Data^a^ ^,^ ^9^

	**lean loading** $\left[\frac{\mathrm{mo}l_{CO_{2}}}{\mathrm{mo}l_{\mathrm{AMP}}+\mathrm{mo}l_{\mathrm{PZ}}}\right]$			**Specific Reboiler Duty [** $\frac{\mathrm{MJ}}{\mathrm{kg}\,\;\mathrm{of}\,\;CO_{2}}]$		
run	experimental	simulation	absolute relative deviation [%]	experimental	simulation	absolute relative deviation [%]
G1	0.019	0.012	34.6	6.61	6.63	0.4
G2	0.091	0.128	40.3	2.92	3.30	13.0
G3	0.051	0.071	38.7	3.13	3.37	7.6
G4	0.192	0.216	12.3	3.10	3.37	8.6
G5	0.224	0.216	3.8	3.58	3.42	4.5
G6	0.214	0.210	1.8	3.65	3.60	1.5
G7	0.217	0.215	0.9	4.23	4.19	1.0
G8	0.011	0.019	71.4	5.00	5.21	4.1
G9	0.084	0.083	0.9	3.23	3.27	1.3
G10	0.071	0.052	27.4	3.34	3.30	1.1
G11	0.131	0.137	4.5	3.32	3.46	4.1
G12	0.168	0.163	3.3	3.51	3.44	2.1
G13	0.205	0.192	6.2	3.83	3.58	6.5
G14	0.210	0.191	9.0	3.98	3.57	10.3
G15	0.221	0.225	1.8	3.58	3.65	2.0
G16	0.202	0.194	3.8	3.86	3.70	4.0
G17	0.205	0.181	11.8	4.02	3.62	10.0

a The absolute relative deviation is calculated as $\frac{\left|y_{\mathrm{mod}el}-y_{\exp erimental}\right|}{y_{\exp erimental}}\times 100$

The ratio between the predicted and experimental CO_2_ capture rates for the different cases for the stripper validation was analyzed similarly to what has been done for the absorber validation. No systematic error trend was detected, as shown in Figure S6. Three different types of temperature profiles are observed in the stripper section. Figure 11 shows the model prediction of the temperature profile for the three different cases. For the experimental case G8, the pinching happens at the bottom of the column. This profile takes place at very low lean loading (G1, G8), where the ratio between the vapor and liquid stream is high enough to make the stripping happen as soon as the rich liquid enters the absorber. In case G10, the temperature profile develops along the whole stripper, indicating that the desorption takes place along the whole column; this happens for the cases with moderate lean loading (G3, G9, G10). In case G15, a temperature pinch happens at the top of the column; this profile takes place at relatively high lean loading (G2, G4, G5, G6, G7, G11, G12, G13, G14, G15, and G17). For these cases, the stripping mainly takes place in the reboiler. Blauwhoff et al.^57^ described the existence of two different operational regimes in the desorber: the heat-limited regime and the stripping-limited regime. In the heat regime, the amount of energy is determined by the sensible heat, desorption heat, and latent heat. In the stripping-limited regime, the amount of steam is determined by the minimum amount of vapor necessary to provide enough driving force for the desorption to happen. The cases where the pinching happens at the bottom of the column belong to the stripping-limited regime, and the cases where the pinching happens at the top belong to the heat-limited regime. The model accurately predicts the different kinds of temperature profiles in the stripper. The predicted temperature profiles in the stripper for the other cases are available in the Supporting Information, Figure S5.

**Figure 11:**
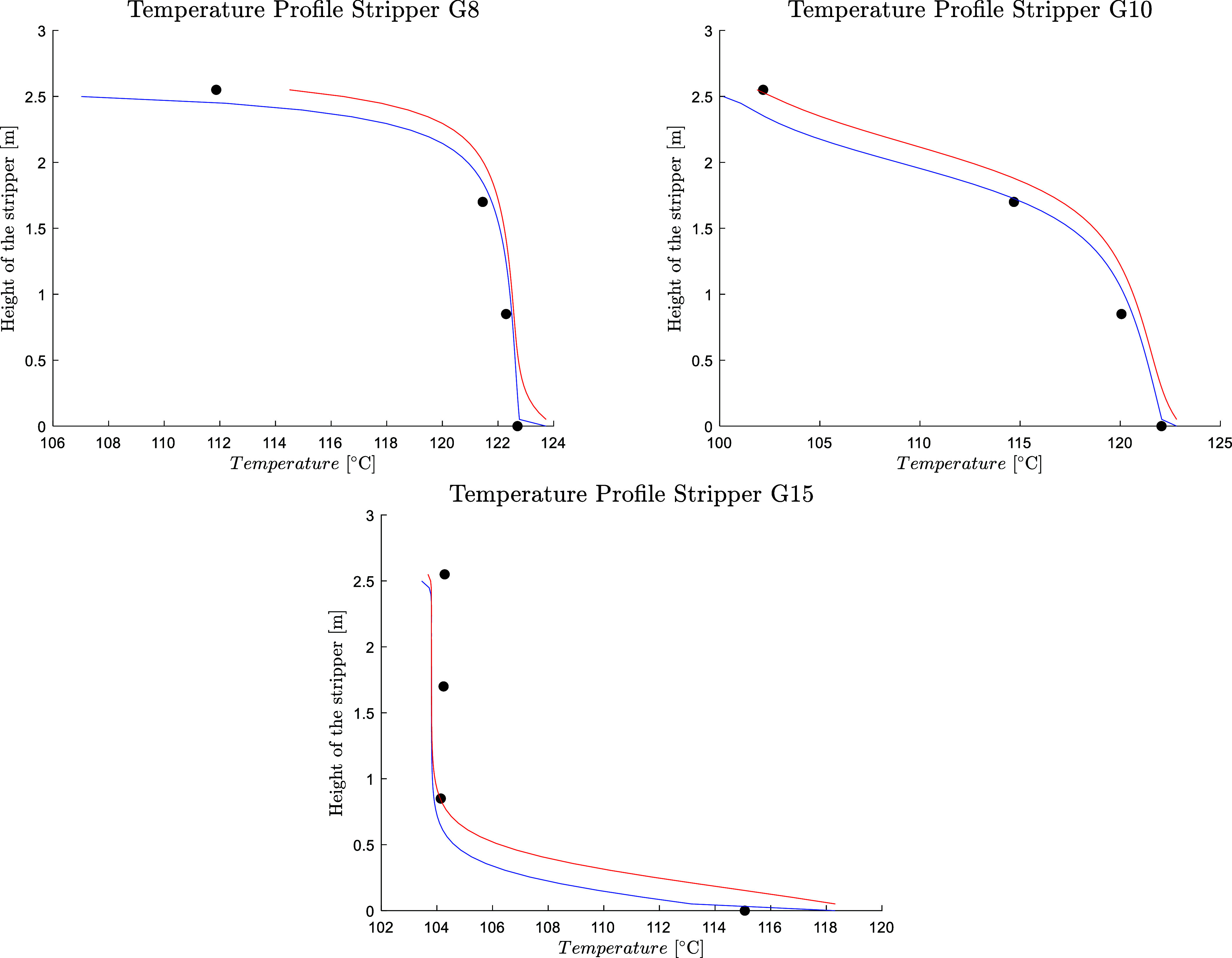
Temperature profile along the stripper (blue line, liquid temperature; red line, vapor temperature; circle solid, experimental data) for three selected cases from Mangalapally.^9^

### Technology Centre of Mongstad (TCM): Absorber Validation

The experimental results of CO_2_ loading in the rich stream leaving the absorber, the experimental CO_2_ capture and the simulated results for the TCM campaign are reported in Table 8. The model predicts the rich loading and the CO_2_ removal within an absolute average relative deviation (AARD) of 3.6% and 4.6%, respectively. For all the cases, the CO_2_ capture rate is predicted within a 5.0% error except for case A5, where a deviation of 17% is observed. However, the rich loading is predicted within a 6.4% error, which may indicate a low accuracy in the closure of the mass balance for this case, as also noted by Morgan et al.^8^ If we exclude case A5 in the error calculation, the CO_2_ Removal AARD is 2.5%, and it gets comparable to the model deviation for the Kaiserslautern plant.

**Table 8:** Absorber Simulation Results (CO_2_ Rich Loading and CO_2_ Removal) for Validation of the TCM Campaign[Table-fn t8fn2] ^,^ ^8^

	**rich loading** $\left[\frac{\mathrm{mo}l_{CO_{2}}}{\mathrm{mo}l_{\mathrm{AMP}}+\mathrm{mo}l_{\mathrm{PZ}}}\right]$			**CO_2_ removal [%]**		
run	experimental	simulation	absolute relative deviation [%]	experimental	simulation	absolute relative deviation [%]
C5	0.539	0.531	1.4	90.37	86.27	4.5
D5	0.422	0.426	1.0	97.73	97.55	0.2
E1	0.419	0.431	2.8	90.69	93.68	3.3
F4	0.535	0.523	2.1	90.16	86.85	3.7
BB3	0.544	0.502	7.7	98.44	99.59	1.2
A5	0.608	0.570	6.4	90.37	74.86	17.2
AA2	-	0.510		92.31	94.09	1.9

a The absolute relative deviation is calculated as $\frac{\left|y_{\mathrm{mod}\mathrm{el}}-y_{\exp\mathrm{erimental}}\right|}{y_{\exp\mathrm{erimental}}}\times 100$

No systematic error has been detected as a function of the rich and lean loading, liquid to gas mass ratio, and packing height, Figure 10.

## Conclusions

In this work, a new e-NRTL framework for the absorption of CO_2_ in aqueous AMP and AMP/PZ solutions is provided. The proposed e-NRTL AMP/H_2_O/CO_2_ model predicts the CO_2_ partial pressure, *P*_CO_2__, with an AARD value of 20.7%, the total pressure of the system with an AARD value of 10.4%, and the heat of absorption of CO_2_ with an AARD value of 11.5%. The model gives a good representation of the liquid speciation of the solvent based on the available experimental data. The proposed e-NRTL AMP/PZ/H_2_O/CO_2_ model predicts *P*_CO_2__ with an AARD value of 26.3%, the total pressure of the system with an AARD value of 7.0%, the heat of absorption of CO_2_ with an AARD value of 10.2%, and the estimated free CO_2_ concentration with an AARD value of 13.1%. The model gives a good representation of the liquid speciation as a function of the CO_2_ loading and amine concentration.

The developed thermodynamic model, in combination with mass transfer and CO_2_ absorption kinetics modeling, was validated using pilot campaigns at the University of Kaiserslautern and one at the Technology Centre of Mongstad (TCM). The two pilot plant campaigns cover a wide range of operative conditions and provide valuable data sets for process model validation. Considering the absorber, the developed rate-based model predicts the CO_2_ rich loading, CO_2_ removal for the TCM campaign with an AARD of 3.6% and 4.6%, respectively. Further, the model developed predicts the CO_2_ rich loading, CO_2_ removal for the Kaiserslautern campaign with an AARD of 1.8% and 2.0%, respectively. For the Kaiserslautern campaign, the model accurately predicts the temperature profile and the CO_2_ liquid concentration profiles along the absorber column. The stripper column was validated using the pilot campaign conducted at the University of Kaiserslautern. The model predicts the specific reboiler duty with an AARD of 4.8%. Furthermore, the model satisfactorily predicts the different temperature profiles in the stripper column. Based on the validation results of these two independent campaigns, the absorber and stripper models show no systematic bias to the main process variables. The process model developed in this work is deemed to be accurate for process simulation of the CO_2_ capture process using aqueous AMP/PZ solutions. The model can be used to design the main units in the amine-based process using the CESAR1 technology, to derisk and optimize the process operation.

## Supplementary Material



## Data Availability

The Aspen Plus file containing the model developed in this work is available at the following link: https://esupport.aspentech.com/S_Article?id=000104489.

## Notes

Fitted parameters for the AMP/H_2_O (Table S1), AMP/H_2_O/CO_2_ (Table S2), AMP/PZ/H_2_O/CO_2_ (Table S3–S5) models; validation of the AMP model on other sources (Figure S1); validation of the AMP/PZ/H_2_O/CO_2_ speciation on other sources (Figure S2); predictions of physical properties for the AMP/PZ/H_2_O/CO_2_ system and parameters (Figure S3 and Table S6); absorber and stripper temperature and liquid CO_2_ concentration profile for the Kaiserslautern Pilot Campaign (Figures S4 and S5); error distribution for the stripper validation for the Kaiserslautern Pilot Campaign (Figure S6) (PDF)
