# Supplementary material for: Development of a Validated Rate-Based Model for CO2 Absorption in Aqueous 2‑Amino-2-methyl-1-propanol and Piperazine Blends Using Aspen Plus
Source: Energy Fuels. 2025 Sep 29;39(40):19350–67. doi: 10.1021/acs.energyfuels.5c03281 (PMC12519493; doi:10.1021/acs.energyfuels.5c03281)
Supplement: Supplementary file 1 [file ef5c03281_si_001.pdf]

Supplementary Information

Development of a validated rate-based model for CO_2 absorption in aqueous 2-amino-2-methyl-1-propanol (AMP) and piperazine (PZ) blends using Aspen Plus

Diego Morlando[a], Ying Zhang[b], Shu Wang[c], Hanna K. Knuutila[a]*

[a]Department of Chemical Engineering, Norwegian University of Science and Technology, N-7491 Trondheim, Norway.

[b]AspenTech Shanghai, Pudong, Shanghai 201210, China

[c]Aspen Technology, Inc., Bedford, Massachusetts 01730, United States

*Corresponding Author: hanna.knuutila@ntnu.no

Model Availability

The Aspen file containing the parameters is available for all the AspenTech user at the following link: https://esupport.aspentech.com/S_Article?id=000104489.

Thermodynamic model parameters

Table S1 reported the fitted parameters value and standard deviation for the AMP/H_2O NRTL model.

Table S1: Fitted parameters for the AMP/H_2O system.

Parameter	Component *i*	Component *j*	Value	Standard Deviation
NRTL/1	AMP	H_2O	-0.96332	0.10773
NRTL/1	H_2O	AMP	6.24283	0.25196
NRTL/2	AMP	H_2O	-240.52	39.9655
NRTL/2	H_2O	AMP	-1170.69	87.606

Table S2 reported the fitted parameters value and standard deviation for the AMP/H_2O/CO_2 system.

Table S2: Fitted Parameters for the AMP/H_2O/CO_2 system. *SD not available since the parameters has been fine-tuned manually.

Parameter	Component *i*	Component *j*	Value	Standard Deviation
CPAQ0/1	$AMPH^+$		334565.7	53288.97
DGAQFM/1	$AMPH^+$		-1.8E+08	199867.7
DHAQFM/1	$AMPH^+$		-4E+08	2453479

DGAQFM/1	$AMPCOO^{-}$		-4.7E+08	*
DHAQFM/1	$AMPCOO^{-}$		-7.7E+08	*
GMENCC/1	H_2O	$(AMPH^{+},HCO_3^{-})$	7.139462	0.725344
GMENCC/1	$(AMPH^{+},HCO_3^{-})$	H_2O	-3.67621	0.312398
GMENCC/1	H_2O	$(AMPH^{+},CO_3^{2-})$	6.315688	2.564463
GMENCC/1	$(AMPH^{+},CO_3^{2-})$	H_2O	-3.46588	1.051165
GMENCC/1	$(AMPH^{+},HCO_3^{-})$	AMP	-2.41995	0.246125
GMENCC/1	AMP	$(AMPH^{+},CO_3^{2-})$	1.197928	1.095572
GMENCC/1	$(AMPH^{+},CO_3^{2-})$	AMP	9.734797	20.01135

Table S3 reported the fitted parameters value and standard deviation for the AMP/PZ/H_2O/CO_2 system.

Table S3: Fitted Parameters for the AMP/PZ/H_2O/CO_2 system.

**Parameter**	**Component *i***	**Component *j***	**Value**	**Standard Deviation**
GMENCC/1	H_2O	$(AMPH^{+},PZCOO^{-})$	-2.4924	0.8455
GMENCC/1	$(AMPH^{+},PZCOO^{-})$	H_2O	1.6812	0.6755
GMENCC/1	H_2O	$(AMPH^{+},PZ(COO^{-})_2)$	5.7964	0.5311
GMENCC/1	$(AMPH^{+},PZ(COO^{-})_2)$	H_2O	-3.0092	0.1677
GMENCC/1	AMP	$(AMPH^{+},PZCOO^{-})$	3.4897	31.4733
GMENCC/1	$(AMPH^{+},PZCOO^{-})$	AMP	17.6954	44.1009
GMENCC/1	AMP	$(AMPH^{+},PZ(COO^{-})_2)$	29.4270	281.4326
GMENCC/1	$(AMPH^{+},PZ(COO^{-})_2)$	AMP	0.4212	1.3772
GMENCC/1	AMP	(PZH^{+},HCO_3^{-})	1.7575	5.7998
GMENCC/1	AMP	$(PZH^{+},PZCOO^{-})$	6.0723	84.2791
GMENCC/1	$(PZH^{+},PZCOO^{-})$	AMP	6.4287	28.6347
GMENCC/1	$(PZH^{+},PZ(COO^{-})_2)$	AMP	11.6474	58.3407
GMENCC/1	PZ	$(AMPH^{+},HCO_3^{-})$	17.4751	228.6084
GMENCC/1	$(AMPH^{+},HCO_3^{-})$	PZ	26.1286	3086.9714
GMENCC/1	PZ	$(AMPH^{+},CO_3^{2-})$	-0.7562	1.3499
GMENCC/1	PZ	$(AMPH^{+},PZCOO^{-})$	-6.2081	1.1910
GMENCC/1	$(AMPH^{+},PZCOO^{-})$	PZ	26.8060	1541.6672
GMENCC/1	PZ	$(AMPH^{+},PZ(COO^{-})_2)$	20.0339	345.4805
GMENCC/1	$(AMPH^{+},PZ(COO^{-})_2)$	PZ	-8.6107	0.8653
GMENCC/1	CO_2	$(AMPH^{+},PZ(COO^{-})_2)$	3.3884	53.9677
GMENCC/1	$(AMPH^{+},PZ(COO^{-})_2)$	CO_2	43.7748	12840.0207
GMENCC/1	CO_2	$(AMPH^{+},PZCOO^{-})$	1.0525	8.8932
GMENCC/1	$(AMPH^{+},PZCOO^{-})$	CO_2	4.5911	120.2578
HENRY/1	CO_2	PZ	19.4138	1.8178
HENRY/2	CO_2	PZ	693.8933	588.0011
NRTL/1	$HPZCOO$	AMP	16.1761	21.3534

Table S4 reported the GMENCC parameters while Table S5 the NRTL parameters of the model developed in this work.

Table S4: GMENCC for the AMP/PZ/H_2O/CO_2 model

GMENCC				
**Molecule *i* or Electrolyte *j***		**Molecule *j* or Electrolyte *i***		***Value***
H_2O	-	H_3O^{+}	OH^{-}	8.045
H_3O^{+}	OH^{-}	H_2O	-	-4.072

H_2O	-	H_3O^+	HCO_3^-	8.045
H_3O^+	HCO_3^-	H_2O	-	-4.072
H_2O	-	H_3O^+	CO_3^{2-}	8.045
H_3O^+	CO_3^{2-}	H_2O	-	-4.072
H_2O	-	PZH^+	$PZCOO^-$	1.211
PZH^+	$PZCOO^-$	H_2O	-	-1.294
H_2O	-	PZH^+	$PZ(COO^-)_2$	-3.156
PZH^+	$PZ(COO^-)_2$	H_2O	-	0.000
H_2O	-	PZH^+	HCO_3^-	4.325
PZH^+	HCO_3^-	H_2O	-	1.184
H_2O	-	PZH^+	CO_3^{2-}	0.000
PZH^+	CO_3^{2-}	H_2O	-	0.000
PZ	-	PZH^+	$PZCOO^-$	0.000
PZH^+	$PZCOO^-$	PZ	-	0.000
PZ	-	PZH^+	$PZ(COO^-)_2$	0.000
PZH^+	$PZ(COO^-)_2$	PZ	-	0.000
PZ	-	PZH^+	HCO_3^-	0.000
PZH^+	HCO_3^-	PZ	-	0.000
PZ	-	PZH^+	CO_3^{2-}	0.000
PZH^+	CO_3^{2-}	PZ	-	0.000
CO_2	-	PZH^+	$PZCOO^-$	5.824
PZH^+	$PZCOO^-$	CO_2	-	5.147
CO_2	-	PZH^+	$PZ(COO^-)_2$	2.140
PZH^+	$PZ(COO^-)_2$	CO_2	-	5.157
CO_2	-	PZH^+	HCO_3^-	6.000
PZH^+	HCO_3^-	CO_2	-	5.200
CO_2	-	PZH^+	CO_3^{2-}	5.103
PZH^+	CO_3^{2-}	CO_2	-	5.127
HPZCOO	-	PZH^+	$PZCOO^-$	0.000
PZH^+	$PZCOO^-$	HPZCOO	-	0.000
HPZCOO	-	PZH^+	$PZ(COO^-)_2$	0.000
PZH^+	$PZ(COO^-)_2$	HPZCOO	-	0.000
HPZCOO	-	PZH^+	HCO_3^-	0.000
PZH^+	HCO_3^-	HPZCOO	-	0.000
HPZCOO	-	PZH^+	CO_3^{2-}	0.000
PZH^+	CO_3^{2-}	HPZCOO	-	0.000
H_2O	-	$AMPH^+$	HCO_3^-	7.139
$AMPH^+$	HCO_3^-	H_2O	-	-3.676
H_2O	-	$AMPH^+$	$AMPCOO^-$	8.000
$AMPH^+$	$AMPCOO^-$	H_2O	-	-4.000
H_2O	-	$AMPH^+$	CO_3^{2-}	6.316
$AMPH^+$	CO_3^{2-}	H_2O	-	-3.466
AMP	-	$AMPH^+$	$AMPCOO^-$	8.000
$AMPH^+$	$AMPCOO^-$	AMP	-	-4.000
AMP	-	$AMPH^+$	HCO_3^-	8.000
$AMPH^+$	HCO_3^-	AMP	-	-2.420

AMP	-	$AMPH^+$	CO_3^{2-}	1.198
$AMPH^+$	CO_3^{2-}	AMP	-	9.735
CO_2	-	$AMPH^+$	$AMPCOO^-$	8.000
$AMPH^+$	$AMPCOO^-$	CO_2	-	-4.000
CO_2	-	$AMPH^+$	HCO_3^-	8.000
$AMPH^+$	HCO_3^-	CO_2	-	-4.000
CO_2	-	$AMPH^+$	CO_3^{2-}	8.000
$AMPH^+$	CO_3^{2-}	CO_2	-	-4.000
H_2O	-	$AMPH^+$	$PZCOO^-$	-2.492
$AMPH^+$	$PZCOO^-$	H_2O	-	1.681
H_2O	-	$AMPH^+$	$PZ(COO^-)_2$	5.796
$AMPH^+$	$PZ(COO^-)_2$	H_2O	-	-3.009
AMP	-	$AMPH^+$	$PZCOO^-$	3.490
$AMPH^+$	$PZCOO^-$	AMP	-	17.695
AMP	-	$AMPH^+$	$PZ(COO^-)_2$	29.427
$AMPH^+$	$PZ(COO^-)_2$	AMP	-	0.421
AMP	-	PZH^+	HCO_3^-	1.758
AMP	-	PZH^+	$PZCOO^-$	6.072
PZH^+	$PZCOO^-$	AMP	-	6.429
PZH^+	$PZ(COO^-)_2$	AMP	-	11.647
PZ	-	$AMPH^+$	HCO_3^-	17.475
$AMPH^+$	HCO_3^-	PZ	-	26.129
PZ	-	$AMPH^+$	CO_3^{2-}	-0.756
PZ	-	$AMPH^+$	$PZCOO^-$	-6.208
$AMPH^+$	$PZCOO^-$	PZ	-	26.806
PZ	-	$AMPH^+$	$PZ(COO^-)_2$	20.034
$AMPH^+$	$PZ(COO^-)_2$	PZ	-	-8.611
CO_2	-	$AMPH^+$	$PZ(COO^-)_2$	3.388
$AMPH^+$	$PZ(COO^-)_2$	CO_2	-	43.775
CO_2	-	$AMPH^+$	$PZCOO^-$	1.052
$AMPH^+$	$PZCOO^-$	CO_2	-	4.591

Table S5: NRTL Parameters for the AMP/PZ/H_2O/CO_2 system

Component *i*	Component *j*	Temperature Unit	A_{ij}	A_{ji}	B_{ij}	B_{ji}	C_{ij}
H_2O	CO_2	K	0.000	0.000	0.000	0.000	0.200
H_2O	PZ	K	5.415	1.359	-2105.295	-960.399	0.200
H_2O	HPZCOO	K	0.293	0.075	0.000	0.000	0.300
H_2O	AMP	K	6.243	-0.963	-1170.690	-240.520	0.200
AMP	PZ	K	0.000	0.000	0.000	0.000	0.300
HPZCOO	AMP	K	16.176	21.353	0.000	0.000	0.000
CO_2	AMP	K	0.000	0.000	0.000	0.000	0.300
CO_2	PZ	K	0.000	0.000	0.000	0.000	0.300

AMP/H_2O/CO_2 Additional Validation

Figure S1 shows the validation of the AMP/H_2O/CO_2 model on the CO_2 solubility data by François et al. (2024) at water wash relevant conditions.

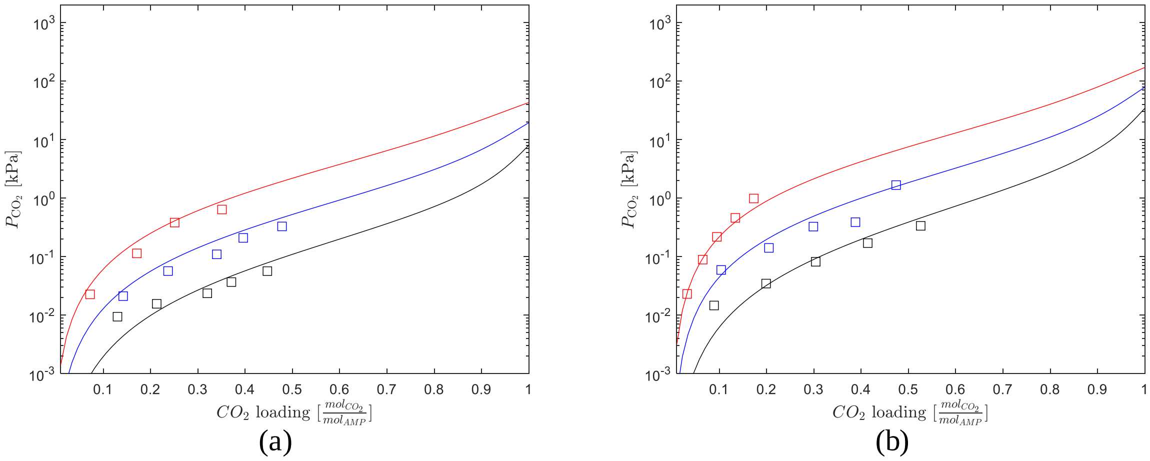

Figure S1: Validation of the AMP/H_2O/CO_2 model on the data by François et al. (2024) at water wash relevant conditions. (a) 0.06 M AMP concentration (b) 0.3 M AMP concentration (Black 40°C, Blue 60 °C, Red 80 °C).

AMP/PZ/H_2O/CO_2 Additional Validation

Figure S2 shows the comparison of the liquid speciation prediction on the experimental data by Li et al. (2014).

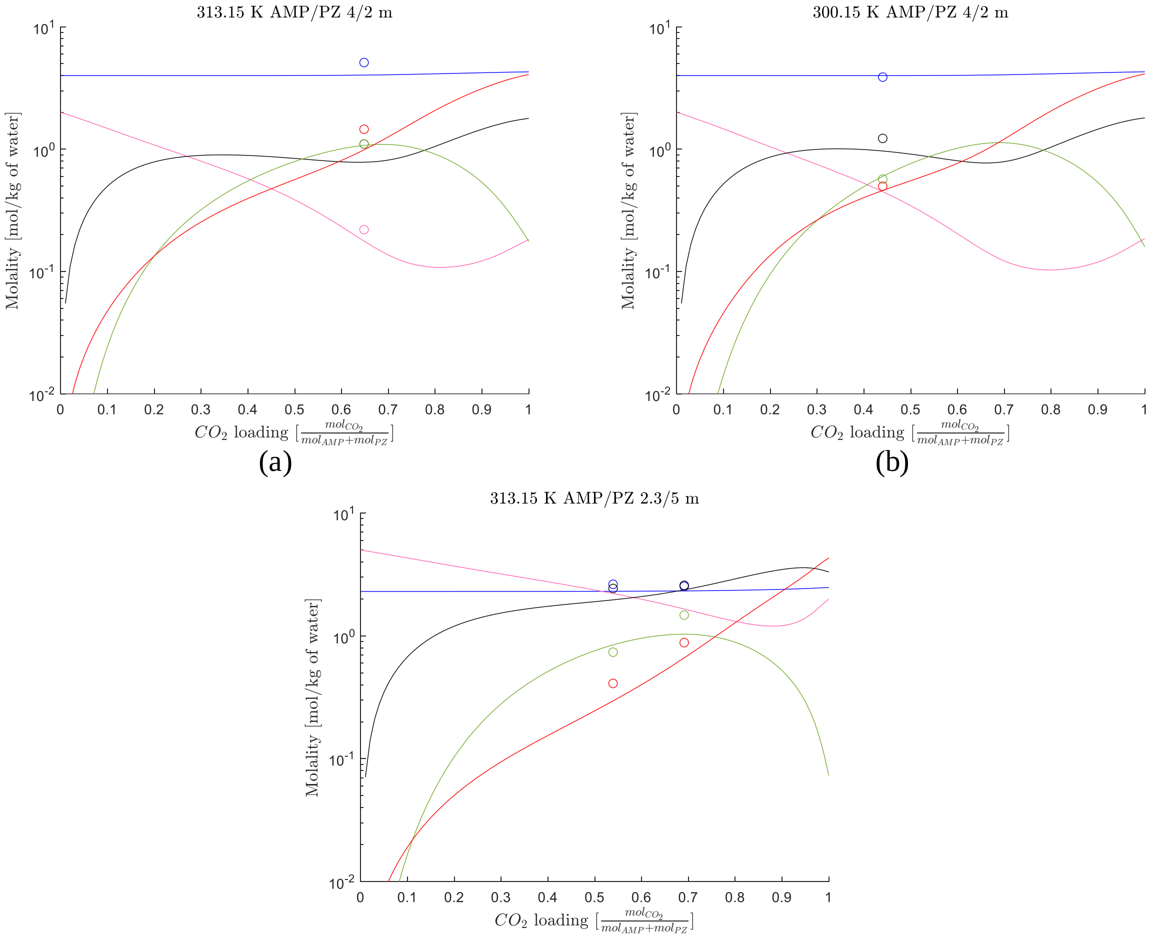

(c)

Figure S2: Model validation on the NMR data by Li et al. (2014), (- blue $AMP/AMPH^{+}$,- purple $AMPCOO^{-}$,- pink PZ/PZH^{+}, - black $PZCOO^{-}/HPZCOO$, - green $PZ(COO^{-})_2$, -red CO_3^{2-}/HCO_3^{-})

AMP/PZ/H₂O/CO₂ Physical Properties

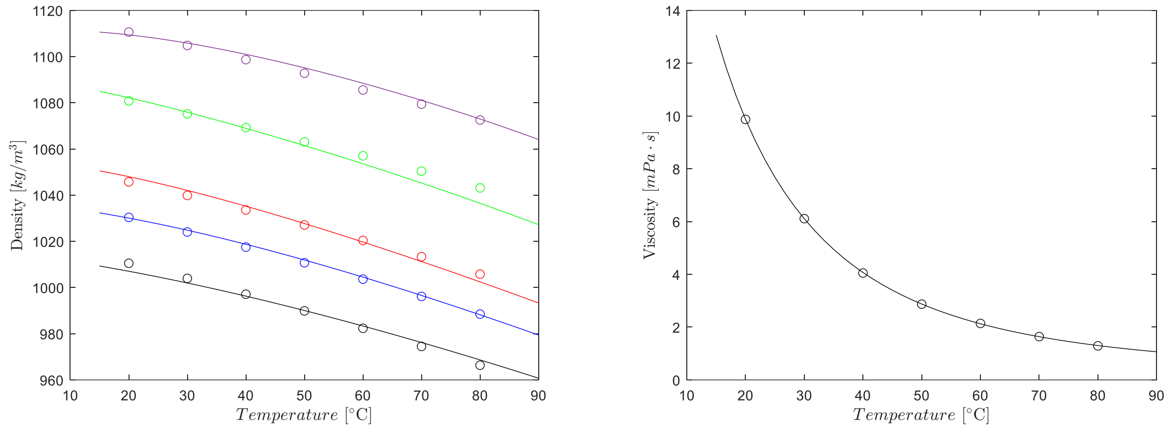

Figure S3: CESAR1 liquid density model prediction on (Morlando et al., 2024) data, (black 0 $\frac{mol_{CO_2}}{mol_{AMP}+mol_{PZ}}$, blue 0.11 $\frac{mol_{CO_2}}{mol_{AMP}+mol_{PZ}}$, red 0.20 $\frac{mol_{CO_2}}{mol_{AMP}+mol_{PZ}}$, green 0.41 $\frac{mol_{CO_2}}{mol_{AMP}+mol_{PZ}}$, purple 0.59 $\frac{mol_{CO_2}}{mol_{AMP}+mol_{PZ}}$). (b) CESAR1 liquid viscosity model prediction on (Morlando et al., 2024) data (black, 0 $\frac{mol_{CO_2}}{mol_{AMP}+mol_{PZ}}$).

**Parameter**	**Component *i***	**Component *j***	**Value**	**Standard Deviation**
VLCLK/1	$AMPH^{+}$	$PZCOO^{-}$	0.16955	0.00158
VLCLK/1	$AMPH^{+}$	$PZ(COO^{-})_2$	0.28325	0.00153
VLQKIJ/1	AMP	PZ	-0.14840	0.07083
MUKIJ/1	AMP	PZ	3.06927	0.95450
MULIJ/1	AMP	PZ	47.90232	2.58465
MULIJ/2	AMP	PZ	45.04628	1.78592

Table S6: Fitted parameters for density and viscosity.

Kaiserslautern Pilot Campaign

**Absorber Temperature Profile**	**Absorber CO_2 Concentration Profile**
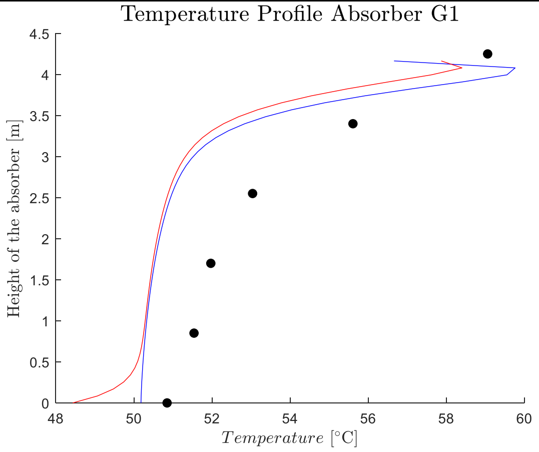	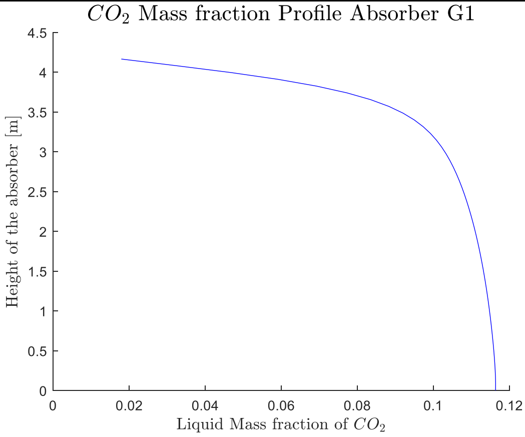

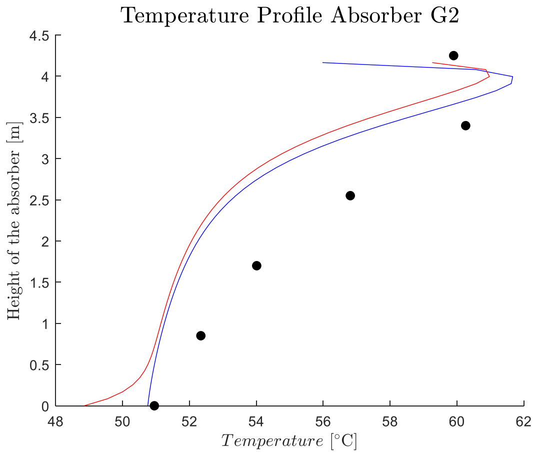
Temperature Profile Absorber G2
Height of the absorber [m]
Temperature [°C]

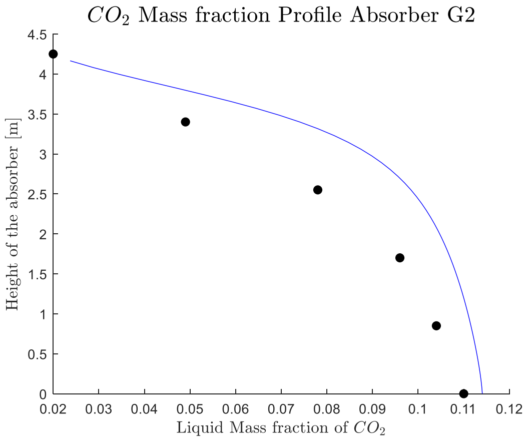
CO_2 Mass fraction Profile Absorber G2
Height of the absorber [m]
Liquid Mass fraction of CO_2

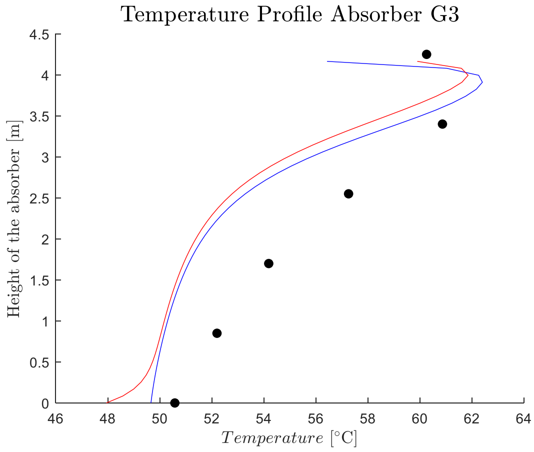
Temperature Profile Absorber G3
Height of the absorber [m]
Temperature [°C]

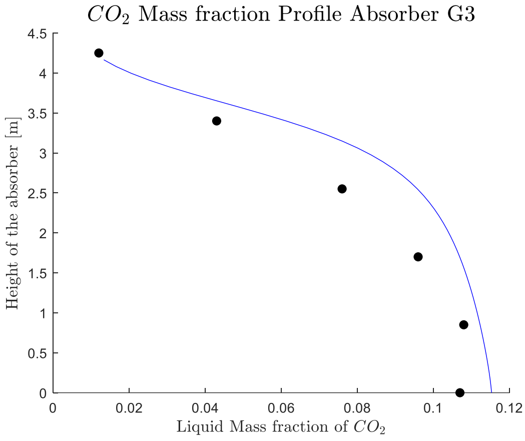
CO_2 Mass fraction Profile Absorber G3
Height of the absorber [m]
Liquid Mass fraction of CO_2

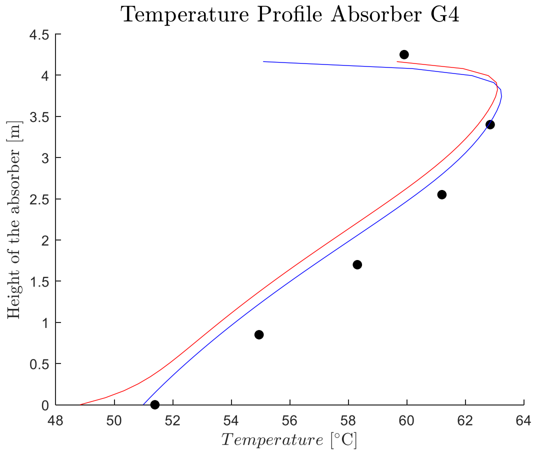
Temperature Profile Absorber G4
Height of the absorber [m]
Temperature [°C]

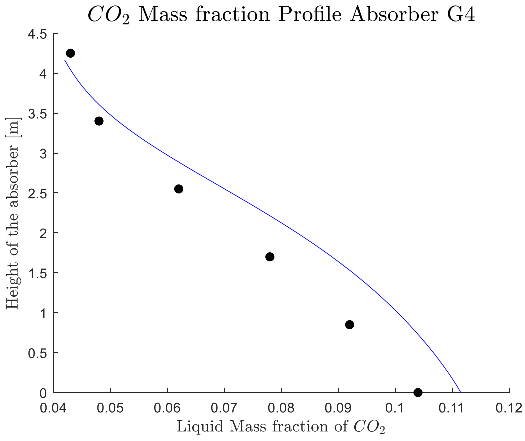
CO_2 Mass fraction Profile Absorber G4
Height of the absorber [m]
Liquid Mass fraction of CO_2

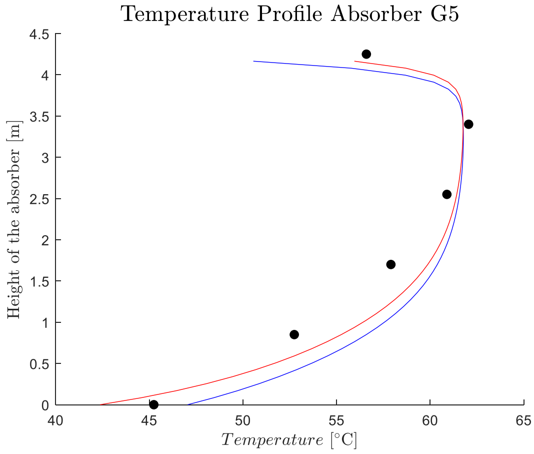
Temperature Profile Absorber G5
Height of the absorber [m]
Temperature [°C]

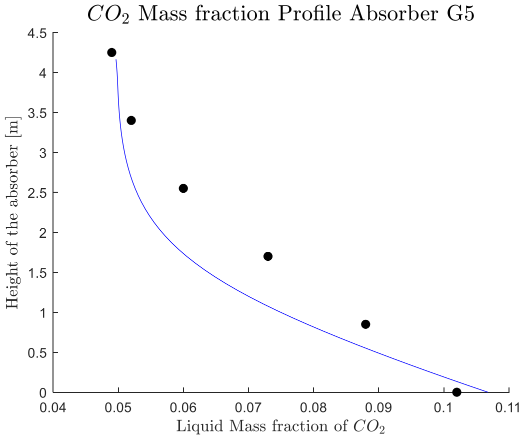
CO_2 Mass fraction Profile Absorber G5
Height of the absorber [m]
Liquid Mass fraction of CO_2

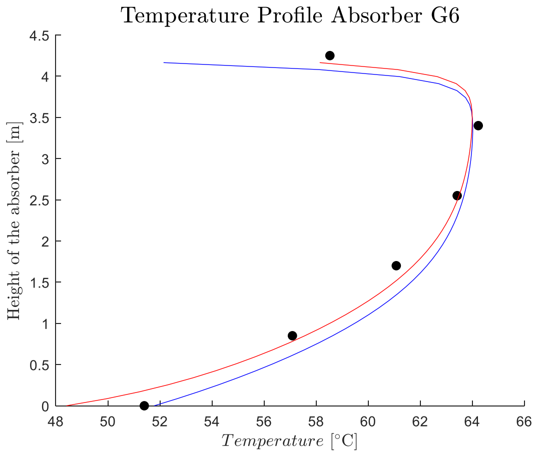
Temperature Profile Absorber G6
Height of the absorber [m]
$Temperature$ [°C]

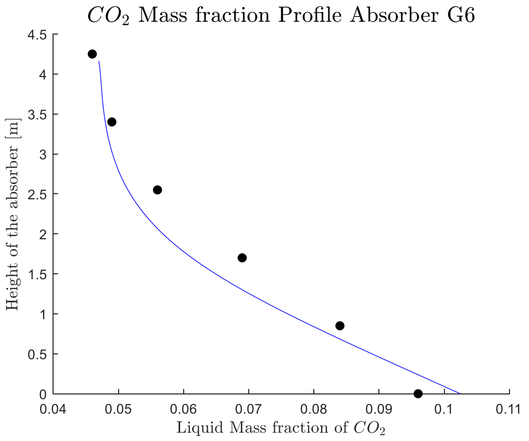
CO_2 Mass fraction Profile Absorber G6
Height of the absorber [m]
Liquid Mass fraction of CO_2

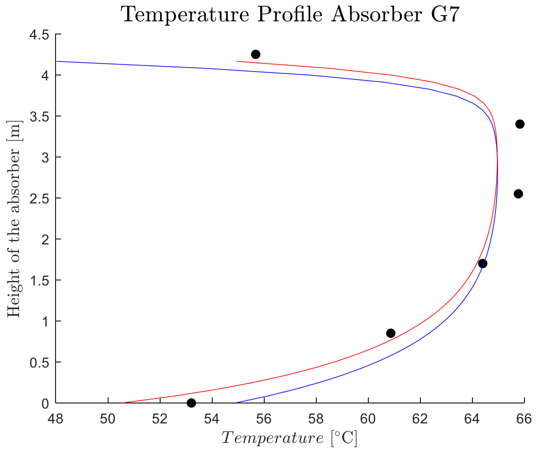
Temperature Profile Absorber G7
Height of the absorber [m]
$Temperature$ [°C]

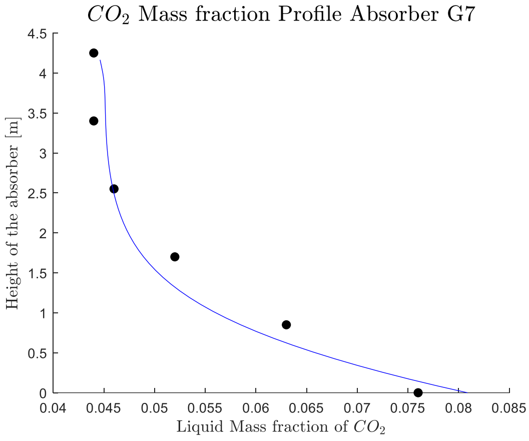
CO_2 Mass fraction Profile Absorber G7
Height of the absorber [m]
Liquid Mass fraction of CO_2

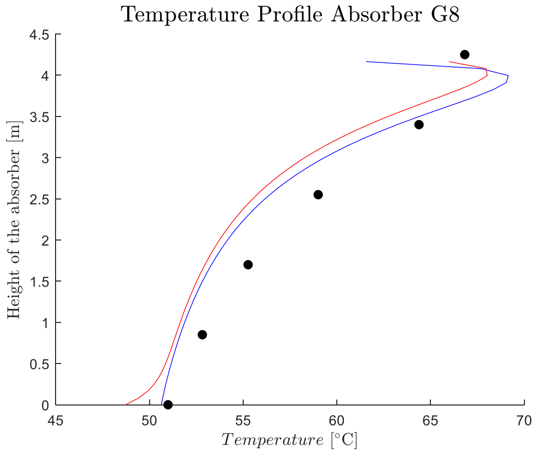
Temperature Profile Absorber G8
Height of the absorber [m]
$Temperature$ [°C]

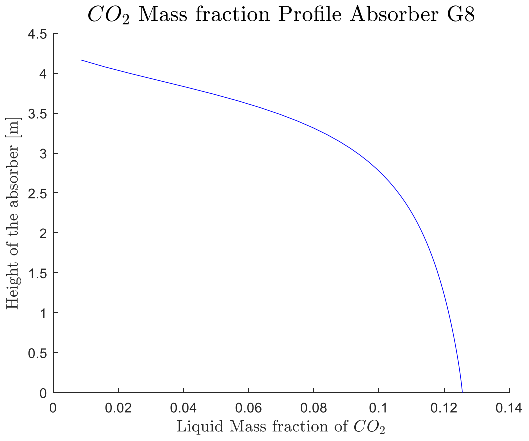
CO_2 Mass fraction Profile Absorber G8
Height of the absorber [m]
Liquid Mass fraction of CO_2

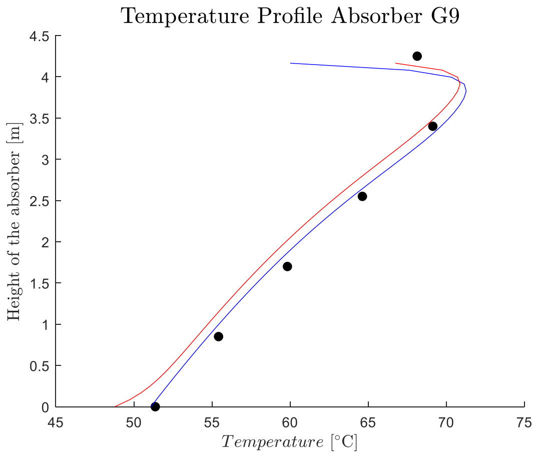
Temperature Profile Absorber G9
Height of the absorber [m]
$Temperature$ [°C]

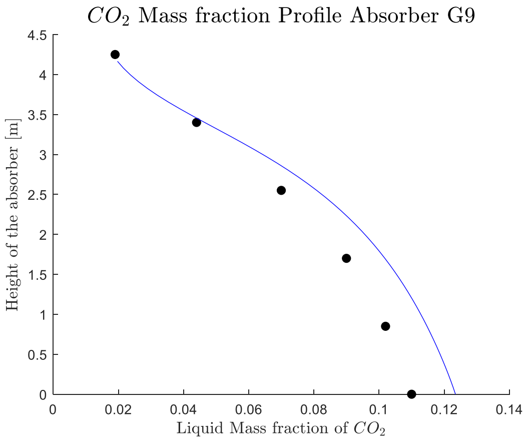
CO_2 Mass fraction Profile Absorber G9
Height of the absorber [m]
Liquid Mass fraction of CO_2

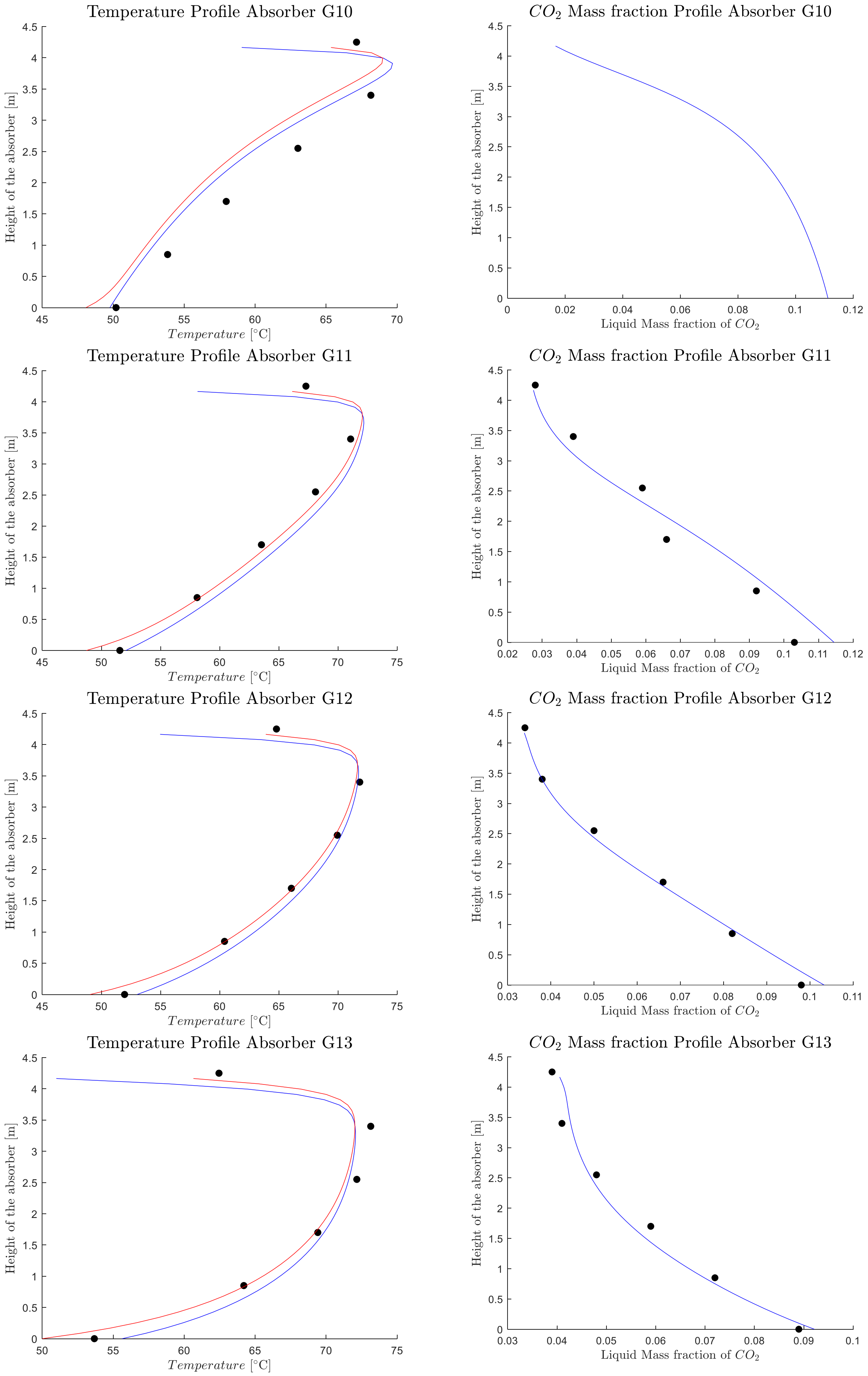
Temperature Profile Absorber G10
Height of the absorber [m]
Temperature [°C]
CO2 Mass fraction Profile Absorber G10
Liquid Mass fraction of CO2
Temperature Profile Absorber G11
CO2 Mass fraction Profile Absorber G11
Temperature Profile Absorber G12
CO2 Mass fraction Profile Absorber G12
Temperature Profile Absorber G13
CO2 Mass fraction Profile Absorber G13

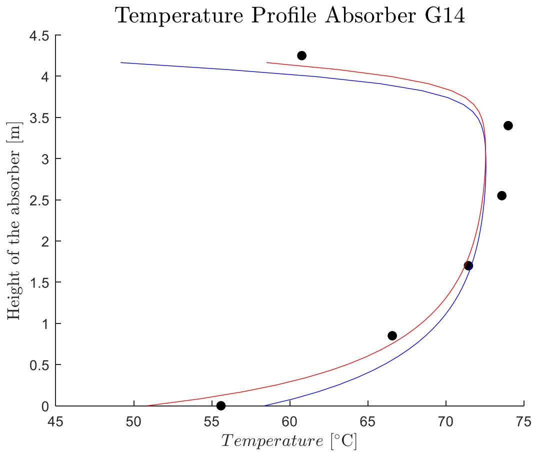
Temperature Profile Absorber G14
Height of the absorber [m]
Temperature [°C]

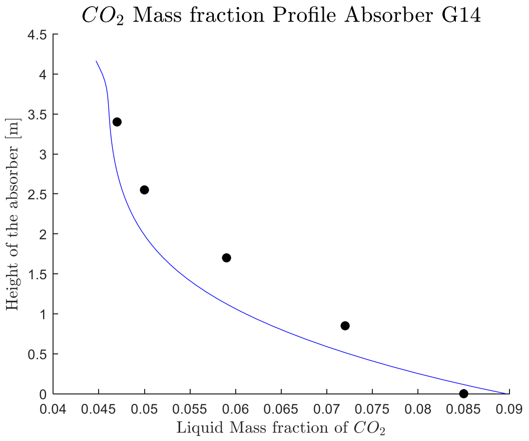
CO_2 Mass fraction Profile Absorber G14
Height of the absorber [m]
Liquid Mass fraction of CO_2

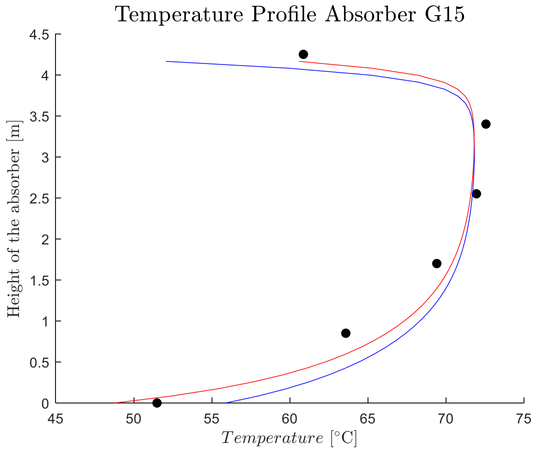
Temperature Profile Absorber G15
Height of the absorber [m]
Temperature [°C]

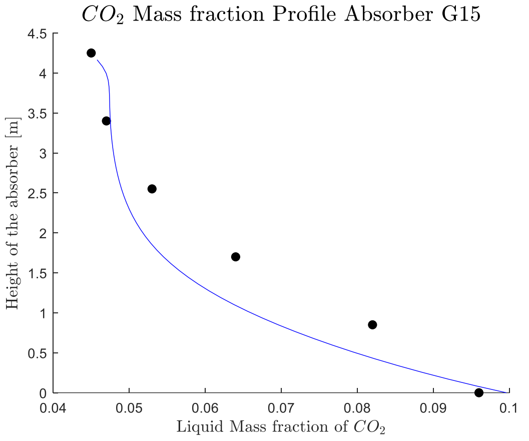
CO_2 Mass fraction Profile Absorber G15
Height of the absorber [m]
Liquid Mass fraction of CO_2

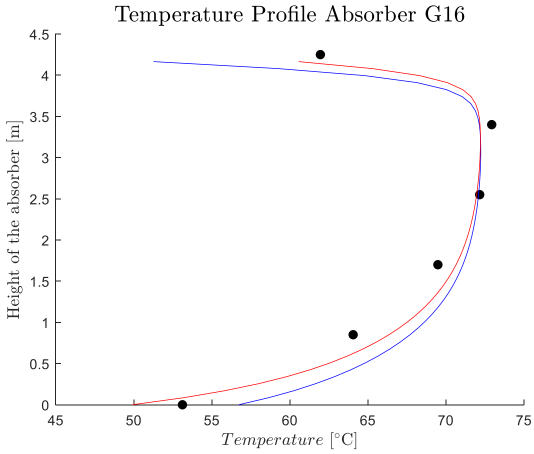
Temperature Profile Absorber G16
Height of the absorber [m]
Temperature [°C]

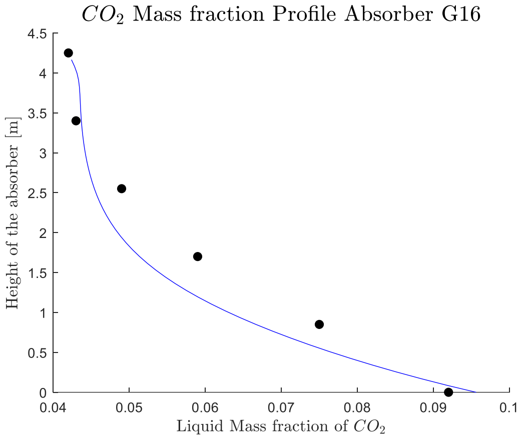
CO_2 Mass fraction Profile Absorber G16
Height of the absorber [m]
Liquid Mass fraction of CO_2

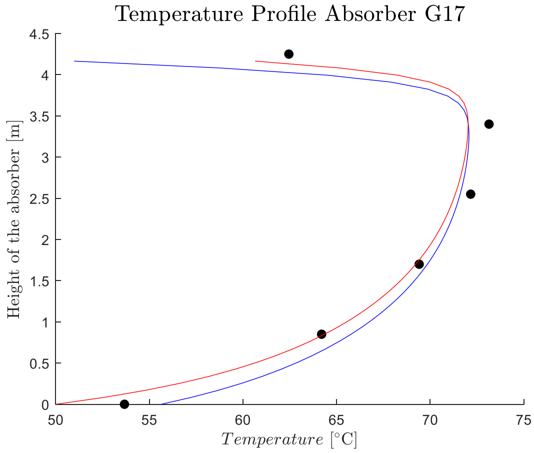
Temperature Profile Absorber G17
Height of the absorber [m]
Temperature [°C]

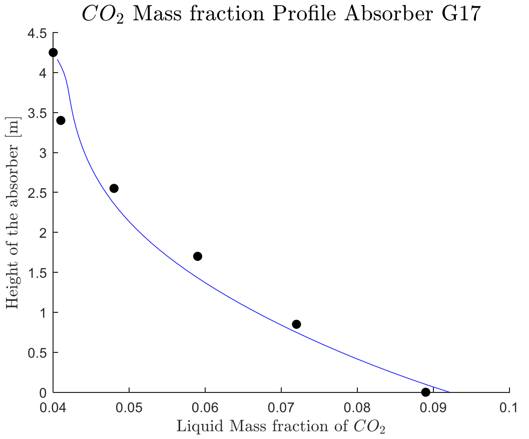
CO_2 Mass fraction Profile Absorber G17
Height of the absorber [m]
Liquid Mass fraction of CO_2

Figure S4: Temperature profile (- blue liquid temperature, - red vapor temperature) and CO_2 concentration profiles in the absorber for the cases at the University of Kaiserslautern (Mangalapally, 2013).

Stripper Temperature Profile

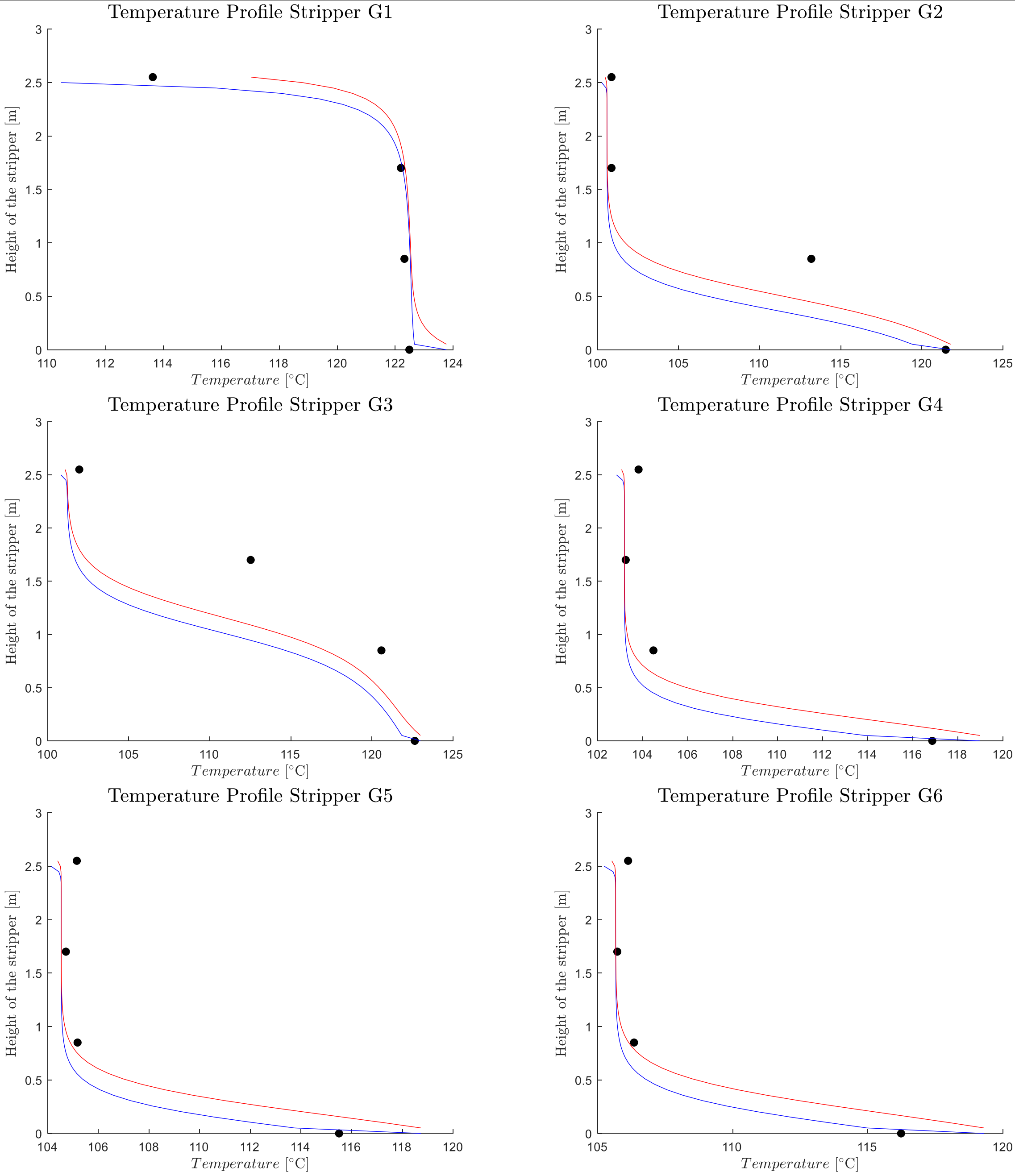

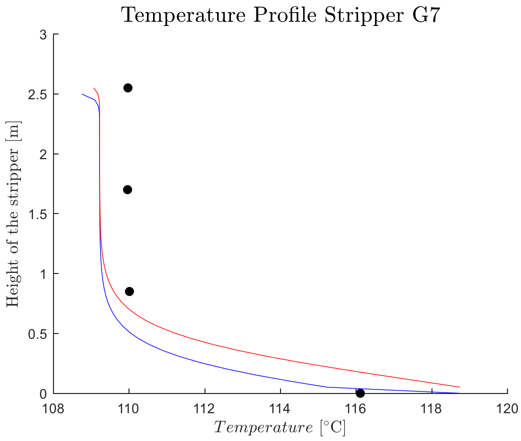
Temperature Profile Stripper G7
Height of the stripper [m]
Temperature [°C]

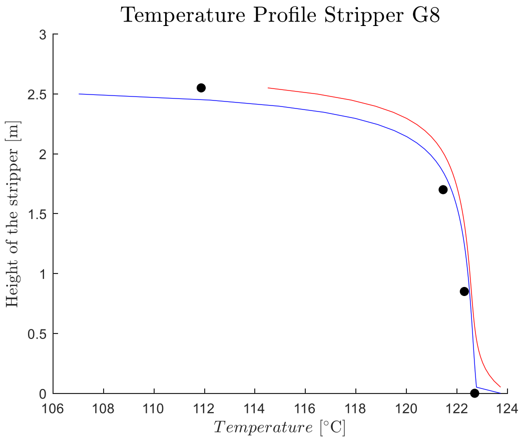
Temperature Profile Stripper G8
Height of the stripper [m]
Temperature [°C]

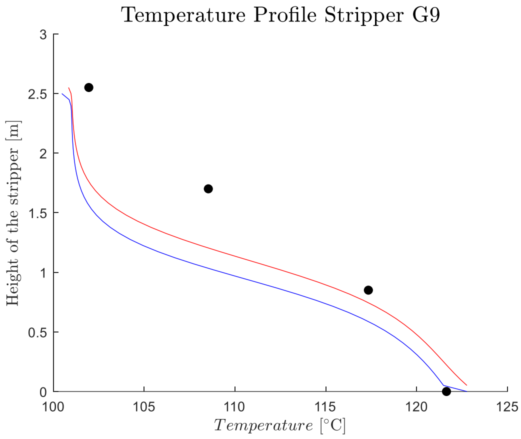
Temperature Profile Stripper G9
Height of the stripper [m]
Temperature [°C]

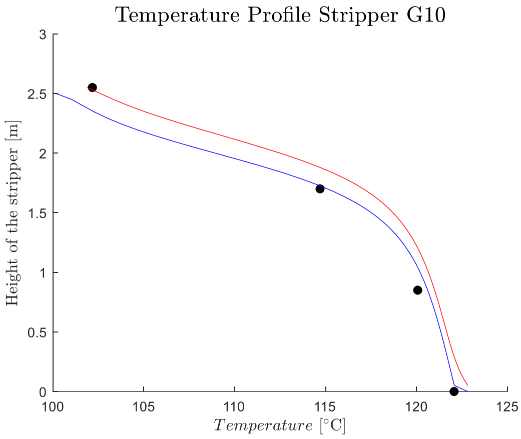
Temperature Profile Stripper G10
Height of the stripper [m]
Temperature [°C]

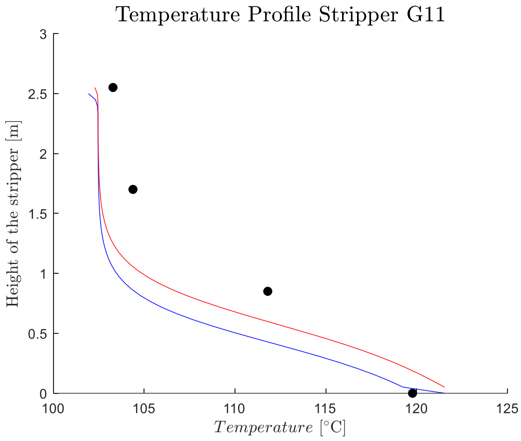
Temperature Profile Stripper G11
Height of the stripper [m]
Temperature [°C]

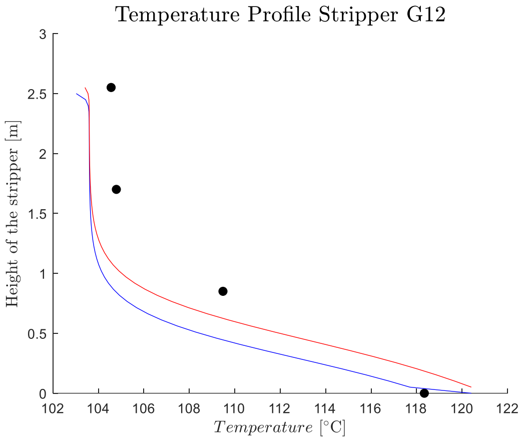
Temperature Profile Stripper G12
Height of the stripper [m]
Temperature [°C]

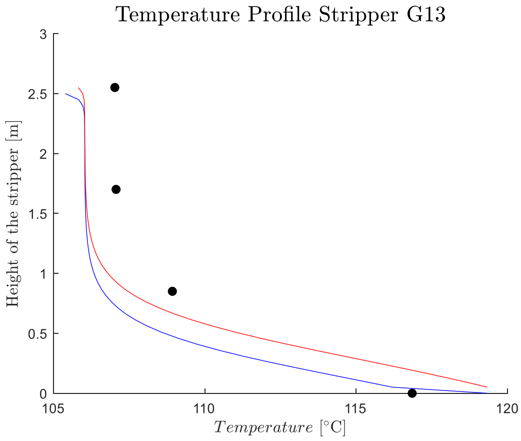
Temperature Profile Stripper G13
Height of the stripper [m]
Temperature [°C]

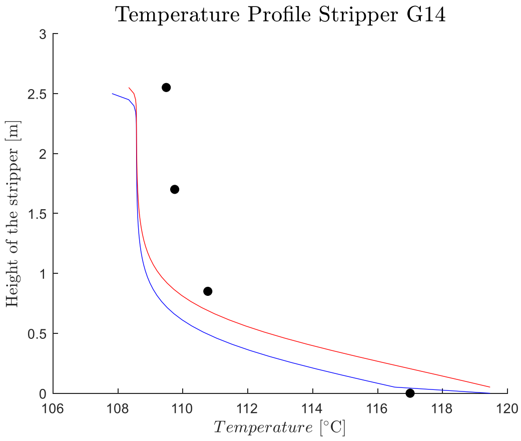
Temperature Profile Stripper G14
Height of the stripper [m]
Temperature [°C]

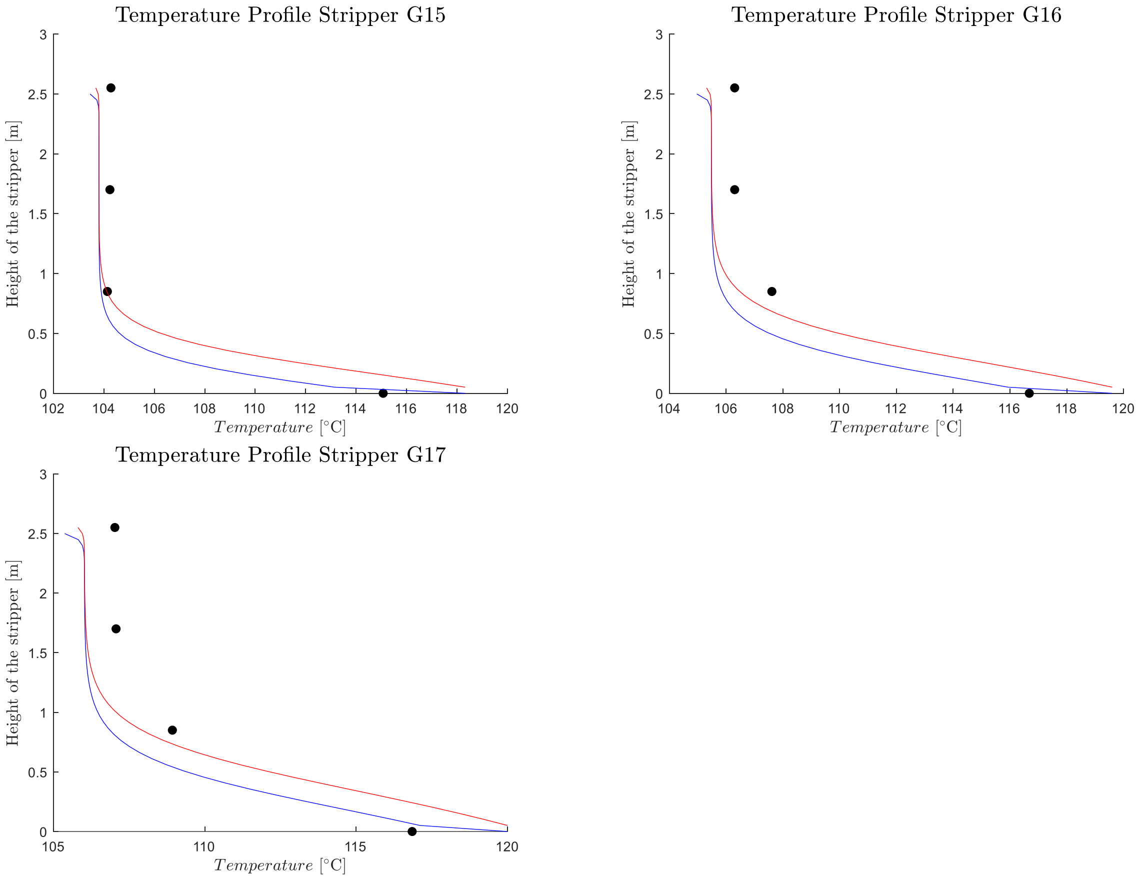

Figure S5: Temperature Profile in the stripper (- blue liquid temperature, - red vapor temperature) for the cases at the University of Kaiserslautern (Mangalapally, 2013).

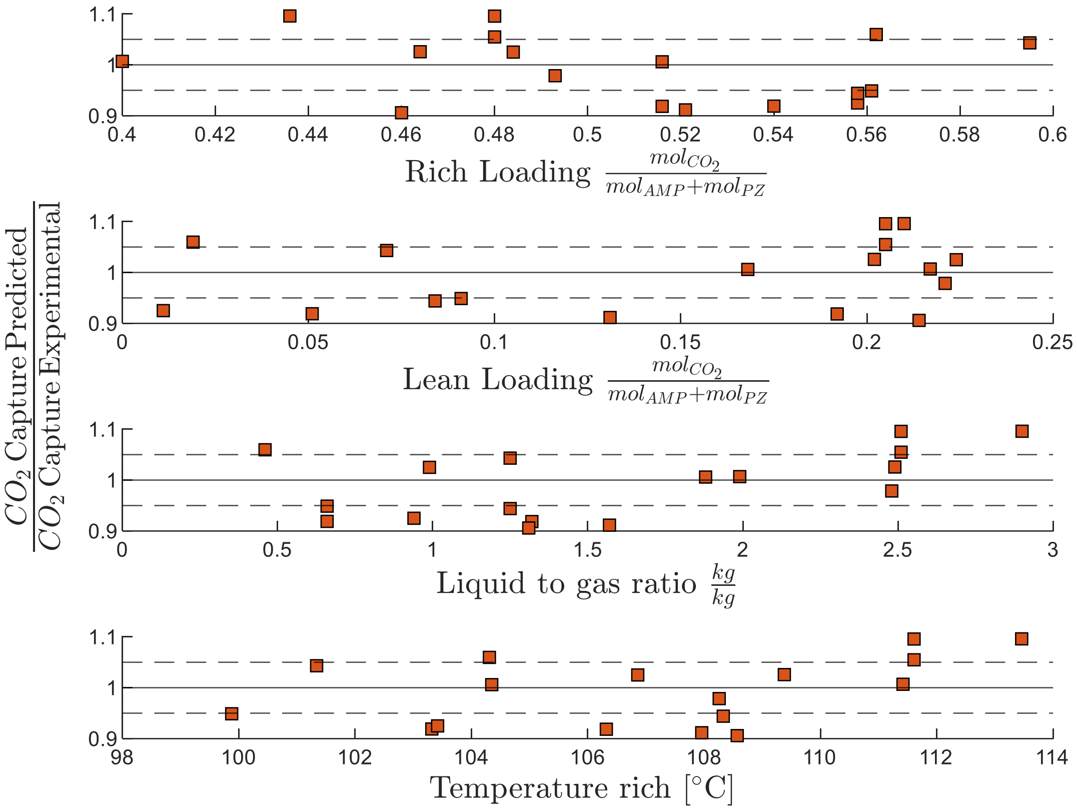

Figure S6: Error distribution for the stripper validation for the Kaiserslautern (Red squares) campaign as a function of experimental rich loading, experimental lean loading, liquid-to-gas ratio and temperature of the rich stream entering the stripper column.

References

François, M. H. J. J., Patil, D., Brulé, S., & Knuutila, H. K. (2024). CO2 solubility and amine volatility data for low-concentration solutions of MEA, AMP, PZ and CESAR-1 blend (AMP/PZ). *Results in Engineering*, *22*, 102163. https://doi.org/10.1016/j.rineng.2024.102163

Li, H., Frailie, P. T., Rochelle, G. T., & Chen, J. (2014). Thermodynamic modeling of piperazine/2-aminomethylpropanol/CO2/water. *Chemical Engineering Science*, *117*, 331–341. https://doi.org/10.1016/j.ces.2014.06.026

Mangalapally, H. P. (2013). PhD Thesis: Pilot plant study of post combustion capture of carbon dioxide with aqueous amine solutions. In.

Morlando, D., Hartono, A., & Knuutila, H. K. (2024). Density and Viscosity of CO2-Loaded Aqueous 2-Amino-2-methyl-1-propanol (AMP) and Piperazine (PZ) Mixtures. *Journal of Chemical & Engineering Data*. https://doi.org/https://doi.org/10.1021/acs.jced.4c00403
